# Exploring therapeutic potential of mitophagy modulators using *Drosophila* models of Parkinson’s disease

**DOI:** 10.3389/fnagi.2022.986849

**Published:** 2022-10-19

**Authors:** Jyotsna Asthana, Bhupendra V. Shravage

**Affiliations:** ^1^Developmental Biology Group, MACS-Agharkar Research Institute, Pune, India; ^2^Department of Biotechnology, Savitribai Phule Pune University, Pune, India; ^3^Department of Zoology, Savitribai Phule Pune University, Pune, India

**Keywords:** Mitophagy, Parkinson’s disease, mitochondria, *Drosophila*, neurodegenerative disorder

## Abstract

Parkinson’s disease (PD) is the second most popular age-associated neurodegenerative disorder after Alzheimer’s disease. The degeneration of dopaminergic neurons, aggregation of α-synuclein (α-syn), and locomotor defects are the main characteristic features of PD. The main cause of a familial form of PD is associated with a mutation in genes such as *SNCA*, *PINK1*, *Parkin*, *DJ-1*, *LRKK2*, and others. Recent advances have uncovered the different underlying mechanisms of PD but the treatment of PD is still unknown due to the unavailability of effective therapies and preventive medicines in the current scenario. The pathophysiology and genetics of PD have been strongly associated with mitochondria in disease etiology. Several studies have investigated a complex molecular mechanism governing the identification and clearance of dysfunctional mitochondria from the cell, a mitochondrial quality control mechanism called mitophagy. Reduced mitophagy and mitochondrial impairment are found in both sporadic and familial PD. Pharmacologically modulating mitophagy and accelerating the removal of defective mitochondria are of common interest in developing a therapy for PD. However, despite the extensive understanding of the mitochondrial quality control pathway and its underlying mechanism, the therapeutic potential of targeting mitophagy modulation and its role in PD remains to be explored. Thus, targeting mitophagy using chemical agents and naturally occurring phytochemicals could be an emerging therapeutic strategy in PD prevention and treatment. We discuss the current research on understanding the role of mitophagy modulators in PD using *Drosophila melanogaster* as a model. We further explore the contribution of *Drosophila* in the pathophysiology of PD, and discuss comprehensive genetic analysis in flies and pharmacological drug screening to develop potential therapeutic molecules for PD.

## Introduction

The incurable progressive neurodegenerative disorder such as Parkinson’s disease (PD) increases with aging and affects the elderly population of age 60 years or above, imposing a huge burden on the welfare of our society. PD is associated with the degeneration of dopaminergic neurons (DA), which diminishes dopamine levels in the substantia nigra and affects the normal function of the brain (Aryal and Lee, 2019). The reduced dopamine level leads to a decline in motor function and movement disorder phenotypes including limb tremors, muscle rigidity, bradykinesia, posture instability, and behavioural disorders like depression and anxiety (Hewitt and Whitworth, 2017). At the cellular level, it is accompanied by the presence of cytoplasmic inclusions called Lewy bodies (LB), which are mainly composed of α-synuclein protein. This protein is considered an executor of PD which plays a prominent role in both familial and sporadic pathogenesis (Fan et al., 2021). The aetiology of PD remains largely unknown and current therapies for PD only alleviate the symptoms of Parkinsonism, and they are not effective in the prevention of PD. Therefore, it is a need of the hour to elucidate the molecular and genetic mechanism of PD pathogenesis for making therapeutic breakthroughs in the future.

**Figure 1:**
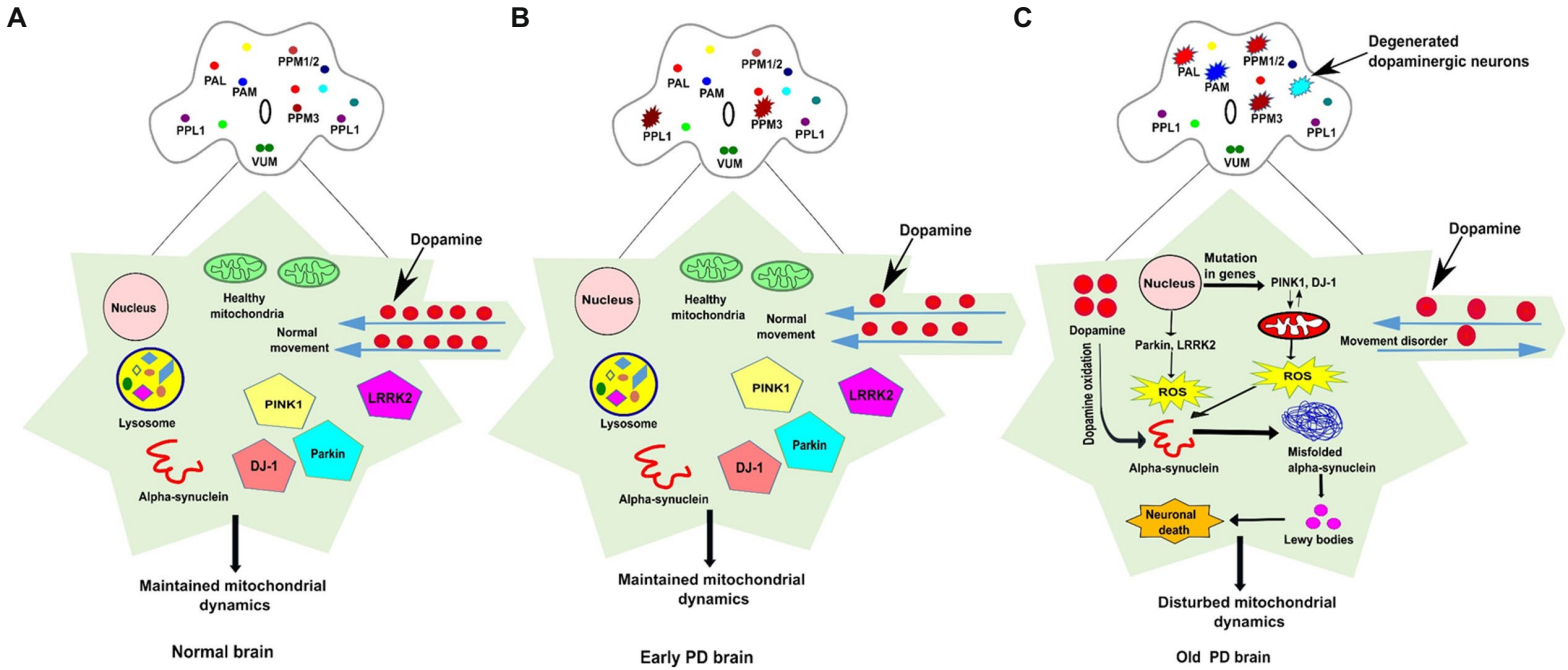
The schematic representation of dopaminergic neuronal clusters and PD-associated genes involved in mitochondrial function in normal, early and old PD affected brains of *Drosophila*. **(A)** The clusters of dopaminergic neurons are distributed all over the *Drosophila* brain. In the normal adult fly brain, healthy mitochondria and *PINK1, Parkin, DJ1,* and *LRRK2* genes maintain the integrity of mitochondrial dynamics. The dopamine movement is found normal, and no loss of DA neurons observed in the healthy brain. **(B)** In the early PD, some (one to four neurons) of the DA neurons found degenerated in the young fly brain but proper PD-associated phenotypes are not observed. **(C)** In PD affected brain, the mutation in genes such as *PINK1* and *DJ-1* stimulate mitochondrial degradation which results in ROS overproduction, whereas dopamine oxidation promotes misfolded α-synuclein aggregation, the formation of Lewy bodies, and induces cellular toxicity which leads to neuronal death. Progressive degeneration of DA neurons (four-six neurons) has been observed in old-aged fly brain. The DA neurons clusters are labelled as PAL-Protocerebral Anterior Lateral; PAM-Protocerebral Anterior Medial; PPM-Protocerebral Posterior Medial; PPL-Protocerebral Posterior Lateral; VUM-Ventral Unpaired Medial; ROS-Reactive oxygen species.

Increasing evidence suggests that the genetic and environmental factors play crucial role in the occurrence of PD, whereas aging is one of the major risk factor amongst these factors. The sporadic and familial are two forms of PD, most of the PD cases are sporadic whereas only 5–10% of cases are familial among all Parkinson’s patients. The genome-wide studies have identified several PD risk loci whose mutations are causative of a familial form of PD which carry heritable changes and disease-associated mutations in “*PARK”* genes (Wakabayashi and Takahashi, 2007). These genes include α-syn (*PARK*1/4) (Polymeropoulos et al., 1997), Parkin (*PARK*2) (Kitada et al., 1998), PINK1 (also known as phosphatase and tensin homolog (PTEN)-induced kinase 1; *PARK*6; Valente et al., 2004), DJ-1 (*PARK*7; Bonifati et al., 2003), LRRK2 (leucine-rich repeat kinase 2-*PARK*8; Zimprich et al., 2004), and ATP13A2 (*PARK*9; Ramirez et al., 2006). The mutations in two genes such as *Parkin* and *PINK1* have been identified in an autosomal recessive form of PD while mutations in both *LRRK2* and *α-syn* result in an autosomal dominant form of PD. Furthermore, three cellular defects such as oxidative stress, mitochondrial dysfunction, and protein aggregation are mainly involved in the progression of this disease. The function of PD-associated genes provides great insights into the development of PD (Figure 1).

DA neurons have significant bioenergetics and metabolic requirements, with the outcomes of acute exposure to mitochondrial stress (Abou-Sleiman et al., 2006). Certainly, mitochondrial dysfunction is known as a prominent pathological hallmark of PD (Fernandez-Moreno et al., 2007). Mitochondria are multifunctional and dynamic organelles contributing to a various range of cellular processes including oxidative phosphorylation, heme biosynthesis, Ca^2+^ signaling, and programmed cell death (Filosto et al., 2011). Mitophagy is a process that maintains mitochondrial homeostasis by degrading superfluous and dysfunctional mitochondria, and impaired mitophagy has been considered one of the crucial aspects in determining pathological variability associated with PD.

Mitophagy involves the accumulation of kinase PINK1 (PTEN-induced putative protein kinase 1) in the outer membrane of depolarized mitochondria where it phosphorylates E3-ubiquitin ligase Parkin which leads to further activation (Geisler et al., 2010; Eiyama and Okamoto, 2015). Activated Parkin subsequently ubiquitinates its substrates in the outer mitochondrial membrane (OMM) including Parkin, TOM70, and SLC 25A4 (Geisler et al., 2010). These polyubiquitinated substrates are identified by autophagy adaptors like p62, NDP52, and OPTN which trigger the binding of Atg8/LC3 and formation of double-membrane autophagosome around the mitochondria (Palikaras et al., 2018a,b). The double layered autophagosome is then transported and degraded in the lysosome, and the degradation products are recycled. Moreover, two important genes, *pink1* and *parkin* are required for mitophagy whereas mutation in these genes can induce PD, suggesting that defective mitophagy may play a role in the development of PD (Durcan and Fon, 2015). Additionally, *pink1* mutation induces defects in the mitophagy process which leads to disruption of cellular and physiological processes including bioenergetics and alteration in the redox state of complex I (Clark et al., 2021). Accumulation of non-functional mitochondria and impaired clearance of such mitochondrial through mitophagy is detrimental to the neurons which trigger their death. Currently, dopamine agonist and monoamine oxidase B (MAO-B) inhibitors are available as preventive medications against PD, however they only alleviate symptoms. Recently, several research groups are focussing their attention on pharmacological screening of chemical/natural modulators which might be useful to promote mitochondrial homeostasis by removing damaged mitochondria and restoring energy homeostasis within the neurons. To this end, a number of chemical modulators, including curcumin (Van der Merwe et al., 2017), spermidine (Yang et al., 2020), resveratrol (Wu et al., 2018), and Urolithin A (Ryu et al., 2016), have been employed to induce mitophagy and test their effectiveness in preventing neurodegenerative diseases like PD.

The primary reason for the utility of model systems in modern biology is the evolutionary principle that all organisms share a remarkable degree of genetic similarity and developmental processes controlled by genes. Research on the natural course of disease in humans is impossible, unethical, and expensive. The systematic evaluation of efficacy, safety, and study on model organisms is relatively cost-effective, easy to maintain, and they can also be experimentally manipulated in research laboratories. Thus, model organisms provide rapid and novel insights into neurodegenerative diseases including PD that can be useful in the era of modern medicine.

Studies on PD have been conducted in a variety of model organisms including mice, fruit flies, worms, and *in vitro* using cell culture (Breger and Fuzzati Armentero, 2019), due to the limitations of working on humans as described above. Indeed, genetic or mitochondrial toxin-based cellular and animal PD models have been utilized to fully understand the pathophysiology of PD, although cellular models have drawbacks as well (Betarbet et al., 2002). *Drosophila melanogaster*, also known as the fruit fly, has become a potent organism for modelling human neurological disorders, such as PD (Feany and Bender, 2000). The fruit fly is a simple animal with a complex neuronal circuit including clusters of DA neurons. The DA neurons can be subjected to genetic manipulations and PD pathogenesis-associated phenotypes have been reproducibly observed in such flies. This model has provided new valuable insights to study the mechanisms underlying PD, and the discovery of novel preventive therapy for neurodegenerative disorders.

So far we have discussed the pathogenesis of PD with a focus on mitophagy. In the next section, we have made an effort to offer a thorough evaluation of the current PD fly models used to investigate disease pathways. In order to clarify altered molecular processes of mitophagy pathways in PD fly models, we will also explore the function of various mitophagy modulators (chemical/natural). Finally, we will discuss previous studies for developing therapeutic strategies against PD by targeting genetic or pharmacological interference of mitophagy pathways.

### *Drosophila melanogaster*: An elegant animal model system for Parkinson’s disease

The common fruit fly, *Drosophila melanogaster*, has been extensively utilized as a genetic model system for investigating molecular and cellular mechanisms underlying disease. The *Drosophila* genome contains homologs of more than 75% of the human disease-causing genes, making them an excellent and practical animal model for improving our understanding of neurodegenerative diseases including PD (Feany and Bender, 2000). *Drosophila* has a short lifecycle of ~10 days at 25°C, and is relatively inexpensive and easily maintained in the laboratory in large numbers. The *Drosophila* genome consists of only four chromosomes as compared to the human genome which is distributed across 23 chromosomes (Lindsley and Zimm, 2012). The UAS-GAL4 binary system has been used as a popular approach for genetic manipulation in *Drosophila*. The UAS-Gal4 system is a binary system: one part encodes GAL4, a transcription factor of yeast (*Saccharomyces cerevisiae*) and the second part consists of Upstream Activator Sequence (UAS) an enhancer to which GAL4 binds (Duffy, 2002). Typically, GAL4 is fused downstream of a tissue-specific promoter (e.g., Tyrosine hydroxylase gene promoter) and flies are engineered to carry this in their genome. Similarly, UAS-transgene (gene of interest, e.g., α-synuclein) is generated and transposed into the *Drosophila* genome. Mating promoter-GAL4 and UAS-transgene flies drive the expression of the transgene in a tissue-specific manner in the DA neurons (Southall et al., 2008).

Over the past decades, different human neurodegenerative diseases have been modeled in *Drosophila* for studying the disease-relevant mechanisms (Bolus et al., 2020). The fact that *Drosophila* has a complex nervous system with neurons and glial cells make the fly model very beneficial for studying the nervous system (Muqit and Feany, 2002). Many animal models have been utilized to study PD, but the fruit flies are a simple animal model in comparison to other model systems. It represents several essential characteristics of PD, including loss of dopaminergic neurons, mitochondrial dysfunction, and degeneration of muscle tissues (Naz and Siddique, 2021). The identification of the SNCA gene was considered to be causative of PD (Kasture et al., 2018). In PD etiology, the importance of associated genes has been frequently rising in the past years. To increase our knowledge in understanding PD etiology, different PD-associated genes have been identified which facilitated the generation of a variety of *Drosophila* models of PD (Hewitt and Whitworth, 2017). The PD-associated genes are functionally conserved across species and associated mechanisms can be easily studied in *Drosophila* models of PD. The *Drosophila* model is excellent *in vivo* system to test potentially neuroprotective compounds which are discussed later.

### *Drosophila* models for Parkinson’s disease

The first *Drosophila* model of Parkinson’s disease was proposed by Feany and Bender in 2000, they established the *Drosophila* model system to study human neurodegenerative diseases. Interestingly, they further described that *Drosophila* α-synuclein model displays locomotion defects and degeneration of dopaminergic neurons similar to PD patients (Feany and Bender, 2000). Even though PD-associated phenotypes observed in the Parkinson’s model have been tested separately, while different laboratories have found conflicting results on the loss of dopaminergic neurons in the fly brain. Hence, all the *Drosophila* models of Parkinson’s disease exhibit different patterns of dopaminergic neuronal clusters in the brain. The fly brain consists of approximately 127 genuine dopaminergic neurons and these neurons are distributed in eight clusters of neurons per hemisphere including 4–13 individual neurons (Kasture et al., 2018).These dopaminergic clusters are named as Protocerebral Anterior Lateral (PAL), Protocerebral Anterior Medial (PAM), Protocerebral Posterior Medial (PPM), Protocerebral Posterior Lateral (PPL), Ventral Unpaired Medial (VUM).

**Figure 2:**
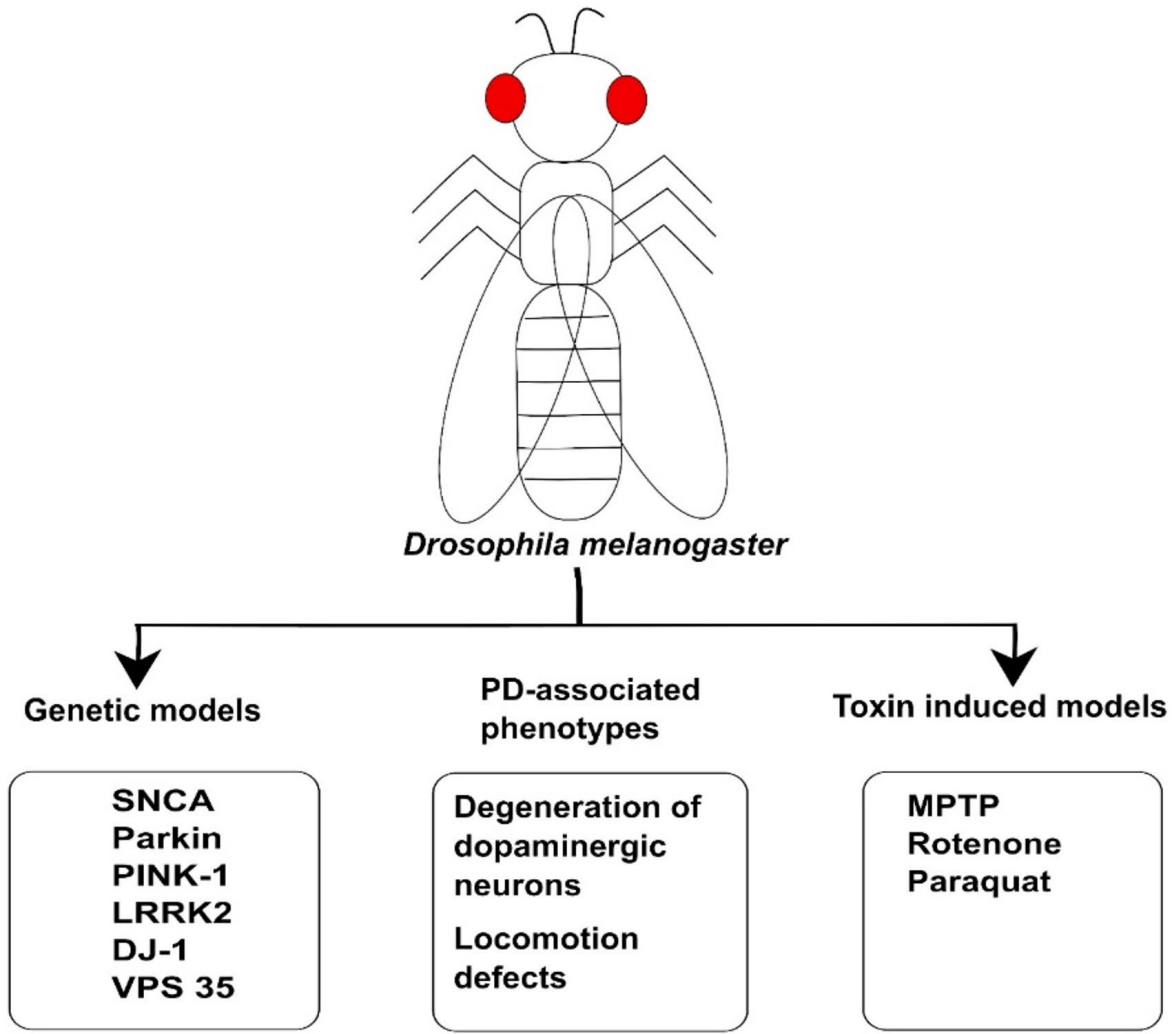
The illustration of Drosophila model used for Parkinson’s Disease. Genetic models such as *SNCA, LRRK2, DJ-1, Parkin*, and *PINK1* have been used to study the molecular mechanism of PD. These animal models have been generated on the basis of genetic mutation in the genes linked to disease. The toxins such as MPTP, Rotenone, and Paraquat have also been used to induce PD-associated phenotypes in *Drosophila* model. These models can also be employed for investigating PD and developing therapeutic drugs against PD.

The discovery of several genes including ***α-synuclein***, ***LRKK2***, ***DJ-1***, ***Parkin***, and ***Pink1*** has offered a novel approach to design different PD models to study the underlying mechanism involved in familial forms of PD (Navarro et al., 2014; Aryal and Lee, 2019). Therefore, different characteristic features and phenotypes have been seen in *Drosophila* model of PD, whether they are produced by mutation in PD-associated genes or exposure to neurotoxin (Figure 2). These features include locomotor defects, reduced lifespan, decrease in dopamine content, and degeneration of DA neurons in the majority of PD models. Therefore, *Drosophila* PD models might be useful for developing new therapeutics to treat PD. The models are individually described below Table 1.

**Table 1:** List of Parkinson’s diseases associated genes and their phenotypic expression in *Drosophila*.

Genetic models for PD	Inheritance	Drosophila melanogaster	Assay used for PD-associated study	Key findings in Drosophila	References
SNCA/PARK1	AD	Expression of Human alpha-synuclein (A30P and A53T) in pan-neurons of larva and adult flies. DA neuronal loss, motor deficits, LB inclusion, and mitochondrial dysfunction.	TH-immunostaining, Lifespan, climbing assay, SEM for adult eye morphology, activated-caspase-3 immunostaining.	Link to retromer and sphingolipids.	Feany and Bender, 2000; Roy and Jackson, 2014
PARKIN/PARK2	AR	Morphological loss of DA neurons, locomotor dysfunction. Lifespan and locomotor ability reduced in KO flies, male sterility.	TH-immunostaining and climbing assay in adults.	Age-dependent mitophagy.	Whitworth et al., 2005
PINK1/PARK6	AR	Loss of DA neurons and locomotor dysfunction and mitophagy of flight muscles with aging in KO mutants.	TH-immunostaining, chemotaxis assay, lifespan, larval crawling assay, HPLC for Dopamine level, RT-PCR for GAPDH2, western blot for TH.	Age-dependent mitophagy, lipid modulation, dysfunction of mitochondrial complex I.	Clark et al., 2006; Park et al., 2006; Yang et al., 2006; Molina-Mateo et al., 2017
DJ-1/PARK7 DJ-1α and DJ-1β	AR	Reduced climbing ability, taste impairment, and defective memory in DJ-1β mutant.	TH- immunostaining, Lifespan.		Meulener et al., 2005; Park et al., 2005
LRRK2/PARK8	AD	RNAi expression of JNK increases *Drosophila* survival time, locomotor activity, and reduces DA neuronal loss in LRRK2G2019S overexpression.	TH-immunostaining, Lifespan.	Link to Rab proteins and autophagy.	Lee et al., 2007; Yang et al., 2018
VPS35/PARK17 GBA Gba 1a Gba 1b	AD AD	Impairment in locomotor activity, disorganization in compound eye and interommatidial bristle loss in VPS35 KD flies. Presence of Dopaminergic neuronal loss and progressive motor defect. GBA gene also involves in the endo-lysosomal pathway and lipid homeostasis	TH-immunostaining, Lifespan TH-immunostaining	Recycling of sphingolipids Developed Parkinsonian signs in *Drosophila* by mutant GBA allele leading to ER stress and death.	Wang et al., 2014; Maor et al., 2016; Sanchez-Martinez et al., 2016
**Toxin-induced models**		**PD-associated phenotypes**					**References**
MPTP/MPP+		Degeneration of DAnergic neurons, loss of DA content, No LB formation, presence of α-synuclein inclusion, reduced motor abilities, No typical PD-associated behaviour observed with acute exposure			Behl et al.,2021;Schober, 2004
Rotenone		Degeneration of DAnergic neurons, LB formation, Parkinsonism, presence of α-synuclein positive inclusion, reduced motor ability			Sherer et al., 2003; Abolaji et al., 2018
Paraquat		LB formation, Degeneration of DAnergic neurons, loss of DA content, accumulation of α-synuclein with long xposure, reduced motor ability			Sherer et al., 2003; Schober, 2004
6-OHDA		No typical LB formation, Loss of DAnergic neurons, Loss of DA content, abnormal motor ability			Schober, 2004;Behl et al., 2021

PD-related genes and their phenotypic expressions in Drosophila melanogaster. PD, Parkinson’s disease; SNCA, α-synuclein; PINK1, PTEN-induced putative kinase 1; LRRK2, leucine-rich repeat kinase 2; DJ-1, protein/nucleic acid deglycase DJ-1; GBA, Glucocerebrosidase; AD, Autosomal dominant; AR, Autosomal recessive; KD, Knockdown; KO, Knockout; DA, dopamine; TH, Tyrosine hydroxylase. Toxin induced models and PD-associated phenotypes; MPTP, 1-Methyl-4-phenyl-1,2,3,6-tetrahydropyridine; 6-OHDA, 6-hydroxy-dopamine; DA, Dopamine; LB, Lewy body; DAnergic; Dopaminergic.

### *SNCA* (α-synuclein)

SNCA encodes a small, self-aggregating, neuronal protein known as α-synuclein. The major component of Lewy bodies in the brain is α-synuclein protein; minor levels of this protein have also been found in heart and muscles. The formation of Lewy body inclusions propelled accumulation and misfolding of α-synuclein in the central nervous system which contributes to dopaminergic neuronal death in PD (Feany and Bender, 2000). Although there is no homolog of SNCA found in *Drosophila*, pathogenic point mutations in SNCA can be expressed in *Drosophila* to generate the GOF model of PD. Several studies have observed that α-synuclein expression can cause different types of phenotypic changes such as a progressive degeneration of dopaminergic neurons and retinal tissues, decline in locomotor ability, and aggregation of proteinaceous inclusions (Auluck et al., 2002; Auluck and Bonini, 2002). However, some discrepancies have been observed related to the strength of α-synuclein phenotypes.

Several studies have suggested that increased proteasome-dependent degradation and pharmacological stimulation of heat shock proteins like HSP70 abolished the α-synuclein mediated toxicity without inducing the formation of inclusion proteins (Auluck and Bonini, 2002; Karpinar et al., 2009; Witt, 2013). Hence, these outcomes indicate an association between increased α-synuclein aggregates and reduced cellular toxicity, supporting *in vitro* evidence that smaller oligomeric species are the primary toxic species in comparison to larger oligomeric species (Karpinar et al., 2009). Numerous recent findings have exhibited intracellular vesicle trafficking by the small GTPase Rab proteins in α-synuclein pathogenesis. In *Drosophila*, the functional interactions between α-synuclein and *Rab1, Rab8a,* and *Rab11* have been identified to ameliorate toxicity (Cooper et al., 2006; Breda et al., 2015; Wang and Hay, 2015; Shi et al., 2017). Notably, both PINK-1 and LRRK2 have also been found functionally associated to *Rab8* but additional studies will be required to investigate the underlying mechanism of Rab proteins in α-synuclein mediated toxicity (Yin et al., 2014; Shi et al., 2017).

There are three PD-associated α-synuclein mutations known as A30P, A53T, and E46K (Blandini and Armentero, 2012). The most studied mutations are A30P and A53T. The transgenic A53T mice exhibited abnormal accumulation of α-synuclein resulting in rapid neurodegeneration which ultimately led to neuronal cell death. The α-synuclein transgenic animals A30P display similar phenotypic and physiological abnormalities of PD including locomotor dysfunction, formation of insoluble fibrillar aggregates, and slow degeneration of dopaminergic neurons as found in humans (Feany and Bender, 2000). Likewise, rapid loss in climbing ability has also been observed in *Drosophila* expressing A30P mutants (Chen et al., 2014). Furthermore, cathepsin D and glucocerebrosidase actively take part in the accumulation of α-synuclein in the brain, resulting in decreased locomotor function along with progressive loss of dopaminergic neurons (Suzuki et al., 2015; Davis et al., 2016; Khair et al., 2018). The human α-synuclein protein contains mitochondrial targeting signals at its N-terminus which plays crucial role in translocating protein to the mitochondrial inner membrane. This α-synuclein accumulation in the mitochondrial membrane of dopaminergic neurons causes increased ROS production, decreased transmembrane potential resulting leading to impairment in mitochondrial function (Devi et al., 2008). A53T α-synuclein mutant phosphorylates Parkin directly at serine-131 to interrupt mitophagy whereas overexpression of A53T α-synuclein mutant affects the activation of p38MAPK (Chen et al., 2018). The accumulation of α-synuclein on mitochondria contributes to increased neuronal death and mitophagy.

### LRRK2

Leucine-rich repeat kinase 2 (*LRRK2*) gene mutations, which involve large domain GTPase and kinase activity, are a frequent cause of autosomal dominant PD. *LRRK2* has been associated to a wide range of cellular processes and signalling pathways, including autophagy, mitochondrial activity, vesicular trafficking, endocytosis, and modulation of the retromer complex (Wallings et al., 2015; Rosenbusch and Kortholt, 2016). The growing evidences suggest that impairment in mitophagy pathway may result from *LRRK2* mutation, but the underlying mechanism is yet unknown. One study claimed that the combined effect of *LRRK2*-G2019S mutation increased the levels of autophagy markers p62 and LC3 in dopaminergic neurons produced from induced pluripotent stem cells from PD patients, showing a link between aberrant autophagy and G2019S-induced neurotoxicity (Liu et al., 2008). However, according to different study, *LRRK2*-G2019S mutations promote mitophagy through activating histone deacetylase (Liu et al., 2019). Further research needs to be carried out to understand these defects at the molecular level. In *Drosophila*, the overexpression of LRRK2 exhibits decreased climbing ability, selective loss of dopaminergic neurons, retinal degeneration, and early mortality (Liu et al., 2008). In addition, JNKK (Hep) was found to mediate LRRK2-induced neuronal degeneration in flies. JNKK is a downstream kinase that can be deleted or expressed in a dominant-negative form to improve locomotion, increase fly survival, and reduce dopaminergic neuron loss in *LRRK2*-G2019S mutants (Yang et al., 2018; Aryal and Lee, 2019).

Numerous studies suggested that *LRKK2* plays a crucial role in PINK1/Parkin-mediated mitophagy. According to the latest study, RAB10 is a substrate of LRKK2 and it aggregates on damaged mitochondria in PINK-1/Parkin dependent manner (Wauters et al., 2020; Singh and Ganley, 2021). However, another study suggested that *LRRK2* can inhibit PINK1 and Parkin-dependent mitophagy clearance through its kinase activity (Bonello et al., 2019; Miller and Muqit, 2019). To shed insight on the function of *LRRK2* in the mitochondrial clearing, additional research is required.

### DJ-1

*DJ-1* is encoded by the *PARK6* gene and mutation in *DJ-1* results in an autosomal recessive PD. *DJ-1* is found in the mitochondrial matrix and intermembrane space, where *DJ-1* binds to mitochondrial complex I subunits and controls its activity (Zhang et al., 2005; Hayashi et al., 2009). Under stress conditions, *DJ-1* translocate into the mitochondria and performs like a redox sensor/reductase. Loss of *DJ-1* (*DJ-1* KO mouse) leads to loss of polarization of mitochondria, mitochondrial fragmentation, and accumulation of markers of autophagy like LC3 punctae around defective mitochondria (Heo et al., 2012). The mitochondrial translocation of *DJ-1* maintains clearance of endogenous ROS, and adenovirus-mediated *DJ-1* overexpression can reverse the mitochondrial fragmentation phenotype of DJ-1 mutant (Menzies et al., 2005; Lucas and Marín, 2007), indicating a critical role for *DJ-1* in mitochondrial function and possible contribution of *DJ-1* in neuroprotection.

A highly conserved protein that is a member of the molecular chaperons superfamily is encoded by *DJ-1*. In *Drosophila*, two orthologues of the *DJ-1, DJ-1α*, and *DJ-1β* are found in the genome (Lucas and Marín, 2007). The predominant expression of *DJ-1α* occurs in the male testis, and adult brain at a lower level. Loss of *DJ-1α* increases oxidative stress and dopaminergic neuronal degeneration, whereas *DJ-1β* mutants exhibit the reduced climbing ability and increased sensitivity against environmental toxins like paraquat, rotenone, and H_2_O_2_ (Menzies et al., 2005; Park et al., 2005; Aryal and Lee, 2019). The oxidative stress and aging process provoke overoxidation of *DJ-1β* at cysteine 104 in *Drosophila*, which inactivates *DJ-1* protein irreversibly (Meulener et al., 2006). Parkin is recruited to damaged mitochondria more readily when *DJ-1* levels are low, and *DJ-1* accumulation on mitochondria under stress circumstances depends on *PINK1* and *Parkin*, demonstrating a link between *DJ-1* and the PINK1/Parkin-mediated mitophagy pathway (Irrcher et al., 2010). The mitochondrial defects in *DJ-1* knockout flies have been demonstrated as phenocopy of PINK1 and Parkin mutants (Joselin et al., 2012). Additionally, the overexpression of *DJ-1* can rescue the phenotypic characteristics of flies that are PINK1 deficient but not in Parkin deficiency.

### Parkin

Autosomal recessive PD is caused by a mutation in the *parkin* gene. *Parkin* encodes an E3 ubiquitin ligase that ubiquitinates substrates to be targeted for proteasomal degradation. Parkin is conserved in *Drosophil*a and loss of *Parkin* leads to loss of mitochondrial integrity (Greene et al., 2003). Despite being partially fatal, homozygous *Parkin* fly mutants are viable and exhibit locomotor defects, male sterility, shortened longevity, mitochondrial dysfunction, degeneration of indirect flight muscles, and dopaminergic neurons (Greene et al., 2003). It has been demonstrated that *Parkin* is essential for preserving the morphological function and integrity of dopaminergic neuronal clusters in the *Drosophila* brain (Cackovic et al., 2018). *Parkin* loss-of-function mutant exhibits accumulation of various Parkin substrates within the brain including DA neurons which may lead to dopaminergic neuronal death (Cha et al., 2005). Interestingly, *Drosophila Parkin* null mutants also demonstrate behavioural phenotype characterized by progressive motor impairment driven by DA neuron degeneration in PPL1 (Protocerebral posterior lateral) cluster and reduced TH staining in PPM1/2 (Protocerebral posterior medial) cluster, resulting in a decreased level of dopamine content (Cha et al., 2005). Taken together, these above studies suggest that *Drosophila parkin* pathological phenotypes are caused by neuronal toxicity and subsequent degeneration.

### PINK1

As like *Parkin*, *PINK1* is also associated with recessive Parkinsonism which encodes a serine/threonine kinase with mitochondrial targeting sequence, and *PINK1* is significantly involved in maintaining the integrity of the mitochondria. In *Drosophila*, *pink-1* mutants exhibit locomotor defects, degeneration of flight muscles and DA neurons, reduced lifespan, and defective thorax phenotype in three-day-old flies (Yang et al., 2006). In addition, aged (30 days old) *pink-1* mutant flies exhibit degeneration of DA neurons in the PPL1 (Protocerebral posterior lateral) cluster. Similarly, *pink-1* mutant caused locomotor defects and degenerated DA neurons in mice (Moisoi et al., 2014). These behavioural phenotypes of the *pink1* mutant were accompanied by mitochondrial dysfunction.

Genetic interaction studies revealed that *pink1* mutant flies share remarkable phenotypic similarities with *parkin* mutants (Clark et al., 2006). *parkin* overexpression rescued phenotypes of *pink1* mutant, while *pink1* overexpression exhibited no effects on *parkin* mutant phenotypes (Park et al., 2006). This genetic analysis indicated that *pink1* and *parkin* function in the same pathway, whereas *parkin* acts downstream of *pink1*, which is conserved between *Drosophila* and mammals. Numerous studies demonstrated that both genes (*pink1* and *parkin*) share a common pathway to maintain mitochondrial integrity, even though their localization is different (Aryal and Lee, 2019). PINK1 localizes primarily to the mitochondria whereas Parkin resides in the cytosol. Furthermore, it has been shown that *parkin* recruitment from the cytosol to depolarized mitochondrial facilitates selective autophagic degradation of defective organelles (Greene et al., 2003; Clark et al., 2006). Additionally, *pink1* along with *parkin* modulates different aspects of mitochondrial functions that also affect its morphology and physiology (Jin and Youle, 2012). Moreover, Parkin is directly phosphorylated by PINK-1 to regulate its translocation to mitochondria in *Drosophila*. These findings explain genetic interactions between *pink1* and *parkin* genes which share a common mechanism in fly model.

### GBA mutations in Parkinson’s disease

*GBA* gene encodes a lysosomal enzyme called glucosidase β-acid/ glucocerebrosidase wherein reduced or null activity of the enzyme leads to accumulation within the lysosome leading to lysosomal dysfunction. Mutation in the *GBA* gene causes an autosomal recessive lysosomal storage disorder known as Gaucher’s disease (GD). Several clinical reports describing GD patients who acquired PD led to the initial hypothesis that GBA mutations and PD are related to each other (Sanchez-Martinez et al., 2016). Furthermore, GBA mutations are considered as strongest risk factor to develop PD and associated phenotypes such as mood disorder and neurons degenerations (Brockmann et al., 2015). Despite being debatable initially, the link between *GBA* mutations and PD is now clear because these mutations are the most prevalent genetic causes of PD globally. Furthermore, this mutation was observed in around 3–20% of the patients in different populations. Based on the kind of GD, GBA mutations are categorised as severe and moderate. This classification correlates with the PD, given that carriers of severe *GBA* mutations have a higher risk than those who have mild *GBA* mutations carriers (Brockmann et al., 2015; Sanchez-Martinez et al., 2016).

Numerous studies have been proposed to explain how *GBA* mutations may cause PD by focusing on loss or gain-of-function mutations, but the precise mechanism is still under investigation. It has been demonstrated that chemical inhibition of GBA can lead to accumulation of SNCA, which was later validated and replicated in other models with *GBA* mutations (Gan-Or et al., 2015). It was also proposed that reduced GBA enzymatic activity (with no genetic lesions in *GBA*) PD patient’s brain is correlated with elevated SNCA. Additionally, SNCA transmission from one cell to another is also accelerated by impaired function of GBA (Gan-Or et al., 2015). A mechanistic suggestion for SNCA accumulation and PD progression is a positive feedback loop where GBA depletion causes further aggregation of SNCA. Apart from this, gain-of-function *GBA* mutation leads to ER stress, which can contribute to the PD progression but is less likely to be a necessary factor in its development (Gan-Or et al., 2018). Null mutations in *GBA* cause PD due to the lack of GBA expression in the neurons. Therefore, if no GBA is produced, there cannot be harmful consequences; instead loss-of-function is more likely to be the cause of elevated risk of developing PD (Velayati et al., 2010).

According to a number of studies, general lysosomal dysfunction and impaired autophagy may result from GBA dysfunction. In addition, the reduced function of GBA was observed in GBA non-mutant PD patients, wherein the GBA levels and its enzymatic activities were found decrease which resulted in reduced lysosomal CMA (chaperon-mediated autophagy) and increased SNCA accumulation (Gan-Or et al., 2015; Behl et al., 2021). Loss of function mutations in GBA can lead to lysosomal dysfunction which can be demonstrated by aggregation of SQSTM1(p62) and polyubiquitinated proteins in a cellular model (Behl et al., 2021). Thus, it may be suggested that factors (genetic or environmental) other than *GBA* mutations may also have an impact on GBA activity, and elucidating these factors might be useful for the understanding of PD aetiology.

### Toxin-induced models of PD

The environmental risk factors and gene–environment interaction play a prime role in sporadic PD development. Numerous researchers have utilized distinct neurotoxins to mimic toxin-induced PD model in *Drosophila*. The most frequently used neurotoxins that lead to a PD phenotype are, paraquat, rotenone, MPTP and 6-OHDA (Schober, 2004). Due to the acute nature of neurotoxin treatment, toxin-induced PD models do not exhibit neuronal loss and protein accumulation in LB. After chronic exposure to neurotoxin treatment, degeneration of dopaminergic neurons and defects in behavioral response was observed in animal models of PD. The above-mentioned toxins except 6-OHDA have been used by various studies in *Drosophila* to model sporadic PD, and provide useful tools for understanding the mechanism of DA neurodegeneration.

### MPTP

MPTP (1-methyl-4-phenyl-1,2,3,6-terahydropyridin) is most commonly used neurotoxin to generate a PD model. Previous neurological studies have reported the absence of Lewy bodies (LB) and severe degeneration of neurons in the substantia nigra of MPTP induced Parkinson’s in humans. MPTP induced models show defects in mitochondrial function which can link mitochondrial complex I inhibition to PD (Hisahara and Shimohama, 2011). MPTP is very lipophilic in nature and crosses the blood brain barrier (BBB) after its systematic administration. Monoamine oxidase B (MAO-B) converts MPTP into 1-methyl-4-phenyl-2,3-dihydropyridium (MPDP+) in glial cells and serotonin neurons, after that MPDP+ oxidizes to MPP+. Subsequently, MPP+ is a polar molecule that is released into extracellular space and cannot transport freely to dopaminergic neurons. Hence, MPP+ requires transporters such as DAT (dopamine transporter) to move it inside the dopaminergic neurons and, it also exhibit high affinity for serotonin and norepinephrine transporters. Thereafter, MPP+ accumulates in mitochondria and impairs their function by inhibiting mitochondrial complex I. The inhibition of complex I enhances the production of ROS in the electron transport chain. Previously, the upregulation of NADPH-oxidase by MPTP was observed in the substantia nigra of mice treated with MPTP. This study also demonstrated that MPTP treatment exhibits progressive degeneration of DA neurons in mice leading to decreased motor abilities (Tieu, 2011). The administration of only high doses of MPTP results in degeneration of dopaminergic neurons in rat, suggesting that it is important to block DA receptors completely for MPTP to exhibit the characteristic features of PD. Furthermore, different animal models have been exposed with MPTP treatment to recapitulate the phenotypic characteristics of a PD. A study reported that natural polyphenol resveratrol extends lifespan and attenuates MPTP-mediated oxidative stress in *Drosophila* (Abolaji et al., 2018). Therefore, resveratrol and other flavonoids can be utilized for developing new therapeutics against PD prevention/treatment. MPTP toxin-induced *Drosophila* model can be a helpful approach in understanding PD and can be used for rapid screening of small molecules including flavonoids and carotenoids that show potential therapeutic activity against PD.

### Rotenone and paraquat

Numerous studies have used mitochondrial complex I inhibitors like rotenone and paraquat to investigate the vulnerability of PD genetic models and their function in neuronal cell death (Sherer et al., 2003; Uversky, 2004). These models also exhibit behavioral and histological changes along with dopaminergic neuronal loss suggesting a pathological role in PD (Trinh et al., 2010; Aryal and Lee, 2019). Rotenone is a pesticide and inhibitor of complex I (found at the inner mitochondrial membrane) which can freely cross cellular membrane and BBB without the support of any transporters. Previous study has shown that chronic exposure to rotenone leads to degeneration of DA neurons and the formation of intracellular inclusions that look like LB in brain tissue (Aryal and Lee, 2019). Rotenone also inhibits cell proliferation and blocks mitosis by perturbing the microtubule assembly and decreasing the rate of GTP hydrolysis (Srivastava and Panda, 2007). Another study has reported that systemic administration of rotenone in rats also leads to PD with symptoms that include changes in behavioral responses and neurodegeneration (Khadrawy et al., 2017).

Paraquat is a well-known nonselective herbicide that induces oxidative stress in cells by increasing the production of cellular ROS. Paraquat can cross the BBB despite its charged chemical structure and causes a significant decrease in the number of dopaminergic neurons in the brain by inducing cell death (Sherer et al., 2003). Long-term exposure to paraquat in mice results in aggregation of α-synuclein in the neurons of substantia nigra. Additionally, the expression of the nicotinic acetylcholine receptor (nAChR) subunit was also found reduced in the presence of chronic exposure to paraquat (Sherer et al., 2003).

### Mitophagy

Mitochondria are dynamic organelle that participates in many cellular functions including, the generation of ATP, regulating cell death, and maintaining ROS homeostasis (Grünewald et al., 2019; Romero-Garcia and Prado-Garcia, 2019). The maintenance of a healthy mitochondrial pool plays a crucial role in cellular and organismal homeostasis. Due to the high energetic demands of neurons, the mitochondrial activity in cells is high, thus making them vulnerable to oxidative damage (Misgeld and Schwarz, 2017; Lou et al., 2020). Therefore, damaged mitochondria need either to be repaired by different mitochondrial quality control mechanisms such as proteasome system, fission-fusion machinery, mitochondrial unfolded response (UPRmt), or to be removed by selective mitochondrial autophagy (Palikaras and Tavernarakis, 2020; Doxaki and Palikaras, 2021). Any kind of impairment in mitochondrial function has been associated with neuronal dysfunction as well as neurodegenerative disorders. Hence, neurons must maintain mitochondrial homeostasis at all times to be able to function optimally.

Mitophagy is a conserved pathway that regulates mitochondrial turnover and is considered an important mechanism for maintaining brain health in higher organisms (Youle and Narendra, 2011; Lou et al., 2020). Impairment in mitophagy may lead to an accumulation of defective mitochondria within cells which cause a variety of pathological conditions including PD (Palikaras et al., 2018a,b; Liu et al., 2019;Vara-Perez et al., 2019; Morales et al., 2020). Numerous mechanisms have been demonstrated to facilitate the removal of damaged mitochondria, highlighting that mitophagy could be activated in response to different stresses through multiple cell signaling pathways in a distinct cellular contexts (Palikaras and Tavernarakis, 2012; Chu, 2019). For simplicity, we have divided the pathways into Parkin-dependent and Parkin-independent mitophagy pathways. PINK-1/Parkin mediated mitophagy pathway is the most studied and well-characterized that orchestrates mitochondrial degradation against stress response.

## Mitophagy mediated pathways

### PINK1/Parkin mediated mitophagy

The PINK1 (PTEN induced putative kinase I)/Parkin pathway is specifically important for maintaining mitochondrial homeostasis. The relationship between PD-associated genes (*pink1* and *parkin*) and mitophagy had been proposed by several researchers in the past few years (Narendra et al., 2008; Pickrell and Youle, 2015). Later, *pink1* and *parkins* were considered as landmark studies of mitophagy and Parkinson’s disease. The mutations in *pink1* and *parkin* results in impaired mitophagy and one of the major factors leading to Parkinson’s disease (Kitada et al., 1998; Valente et al., 2004). Loss-of-function of *PINK1* or *parkin* also leads to accumulation of damaged mitochondria, mitochondrial dysfunction and dopaminergic neuronal loss (Clark et al., 2006; Morais et al., 2014; Liu et al., 2019). Despite recent scientific advancements in this pathway, underlying mechanism of pharmacological modulation of mitophagy remain limited.

PINK1 is a mitochondrial serine/threonine protein kinase encoded by the *PARK6* gene which present on outer surface of mitochondrial membrane. Whereas, Parkin is an E3 ubiquitin ligase encoded by the *PARK2* gene (McWilliams and Muqit, 2017). This pathway involves the assembly of ubiquitin chains for removal impaired mitochondria. Three important elements with different functions such as PINK1 (as a mitochondrial damage sensor), Parkin (as a signal amplifier) and ubiquitin chains (as the signal effectors) collectively make the assembly for mitophagy (Harper et al., 2018). PINK1 is transported to the mitochondria through its mitochondrial targeting sequence (MTS). PINK1 localizes to mitochondrial membrane where it spans across outer (OMM) and inner mitochondrial membrane (IMM) under normal conditions. PINK1 is cleaved and inactivated by the matrix processing peptidase (MPP) and the IMM protease presenilin-associated rhomboid like protein (PARL), and degraded in the cytoplasm by N-end rule pathway (Yamano and Youle, 2013; Sekine and Youle, 2018). However, when the mitochondrial depolarize, PINK1 can no longer be cleaved by MPP and PARL thereby leading to its accumulation in the OMM (Meissner et al., 2015). Here PINK1 undergoes dimerization and self-phosphorylation for its activation. Activated PINK1 subsequently phosphorylates several substrates including Parkin and relieves Parkin from its auto-inhibitory conformation (Narendra et al., 2010; Trempe et al., 2013). Activated Parkin ubiquitinates several proteins and these polyubiquitinated substrates are then recognized by one or more autophagy receptors p62/OPTN/NDP52 (Wong and Holzbaur, 2015). The autophagy receptors in turn interact with Atg8/LC3 through binding to AIR/LIR domain allowing autophagosome formation around the mitochondria ultimately leading to fusion to lysosome and degradation of the contents within mitolysosomes (Harper et al., 2018).

### *Parkin-*independent mitophagy

*Parkin*, an E3 ubiquitin ligase is known as a crucial regulator of mitophagy but several studies suggest that mitophagy can also happen in the absence of *Parkin,* which is called *Parkin*-independent mitophagy. This pathway further divides into two parts such as receptor-mediated and ubiquitin ligase-mediated mitophagy.

## Receptor mediated mitophagy

### BNIP3/NIX (BNIP3L) mitophagy and FUNDC1-mediated mitophagy pathway

BCL2/adenovirus E1B 19 kDa interacting protein 3 (BNIP3) and its homolog Nip3 like protein (NIX) are BH3 only proteins belonging to the BCL-2 family, and both proteins have been shown to regulate cell death (Hanna et al., 2012; Liu et al., 2014; Rogov et al., 2017). Both the proteins translocate on OMM through their C-terminus and their N-terminus has a LIR (LC3 interacting region) motif through which they interact with LC3 or GABARAP (Rogov et al., 2017; Palikaras et al., 2018a,b). Previous studies have shown the involvement of BNIP3 in hypoxia induced mitophagy, where it was shown to promote the translocation of Drp1 and Parkin in cardiomyocytes (Lee et al., 2011; Palikaras et al., 2018a,b). BNIP3 physically interacts with PINK1 and facilitate its insertion into OMM to promote mitophagy, suggesting link between the PINK1/Parkin pathway and receptor-mediated mitophagy during mitochondrial degradation (Liu et al., 2014). Similarly, NIX induces the mitochondrial translocation of Parkin and itself gets ubiquitinated through Parkin (Zimmermann and Reichert, 2018). The crucial role of NIX has also been observed in macrophage polarization and in the development of retinal ganglion cells (Schweers et al., 2007).

In response to specific hypoxia condition, FUN14 domain-containing protein 1 (FUNDC1) acts as a mitophagy receptor by interacting with LC3 to induce mitophagy. FUNDC1 also regulates mitochondrial dynamics through the interaction with Drp1 and Opa1 during hypoxia conditions (Liu et al., 2012). FUNDC1 is phosphorylated by the kinase CK2 (casein kinase II) on Ser13 which is an inhibitory signal while de-phosphorylation of Ser13 by phosphoglycerate mutase 5 (PGAM5) facilitates LC3 recruitment to the mitochondria and mitophagy induction. FUNDC1 can be phosphorylated at serine (Ser17) by ULK1 kinase to increase mitophagy (Palikaras et al., 2018a,b; Zimmermann and Reichert, 2018).

Similar to these mitophagy receptors, other receptors such as AMBRA1, BCL2L13, FKBP8, and prohibitin 2 have been recently identified to induce mitophagy. There are few lipids like cardiolipin that can also bind to LC3 to trigger mitophagy. Under normal conditions, cardiolipin is localized in the IMM, and it is exported to the OMM upon mitochondrial stress and binds to LC3 to trigger mitophagy directly (Chu et al., 2014). Similarly, AMBRA1 possesses a LIR motif and triggers mitophagy induction through PINK1/Parkin pathway. AMBRA1 was found to induce mitophagy independently of PINK1/Parkin pathway under normal condition. Moreover, it was also identified that, AMBRA1 provides protection against ROS production, oxidative stress and promotes mitochondrial clearance in SH-SY5Y neuroblastoma cells (Di Rita et al., 2018). Additionally, other receptors like BCL2L13 (Bcl-2 like protein 13) is an OMM protein which contains a LIR motif and its overexpression caused fragmentation of mitochondria and induce mitophagy (Murakawa et al., 2015). Recently, this protein BCL2L13 has been demonstrated as mammalian orthologue of Atg32 and able to balance for the loss of Atg32 protein in yeast (Palikaras et al., 2018a,b).

Subsequently, FKBP8 (FK 506 binding protein 8) was identified as another mitophagy receptor with an anti-apoptotic role, whereas FKBP8 was not regulated by phosphorylation due to the absence of proper residue near its LIR motif (Bhujabal et al., 2017; Palikaras et al., 2018a,b). The induction of mitophagy was found independent of Parkin upon FKBP8 overexpression.

### Ubiquitin ligase-mediated mitophagy

Previous studies have proposed that mitophagy can also be induced directly (independent of Parkin) by *Pink1* mediated translocation of NPD52 and optineurin to mitochondria, suggesting that Parkin is not essential for mitophagy (Lazarou et al., 2015). Several other ubiquitin E3 ligases have been reported along with Parkin that function in mitophagy. AR1H1, a novel E3 ligase which participate in PINK1 dependent mitophagy (Villa et al., 2017). Mitochondrial ubiquitin ligase (MUL1) activator of NF-κβ1 was proposed to compensate the loss of PINK1/Parkin function and it rescued the associated PD phenotypes (Yun et al., 2014). Taken together, it can be concluded that mitophagy is a complex process and can be regulated be several molecules in Parkin-dependent and independent ways. Hence, it is likely that pharmacological interventions by using flavonoids may trigger one or more pathways of mitophagy induction.

### Pharmacological modulation of mitophagy: From potent modulators to therapeutic strategies

The uncontrolled accumulation of damaged organelles and impaired mitochondrial homeostasis are common denominators of neurodegenerative diseases including PD (Federico et al., 2012; Grünewald et al., 2019). In the post-mortem brains of PD patient, activity of complex I was reduced due to mitochondrial respiratory chain deficit, suggesting an important role of mitochondria in PD pathogenesis (Liu et al., 2019). Additionally, impaired mitochondrial function and reduced mitophagy have been observed as crucial player in determining pathological heterogeneity of brain in PD patients, indicating a connection between mitophagy and PD pathogenesis (Clark et al., 2021). The mutations in most PD associated genes including *PINK1* and *Parkin* participate in mitochondrial dysfunction and mitophagy impairment. Therefore, understanding the underlying mechanisms of mitophagy modulation and how these PD associated genes function to maintain mitochondrial quality control are needed for developing therapeutics against PD.

### Mitophagy modulators

Mitophagy modulators can be chemical or natural molecules that might be used to induce the efficient removal of impaired mitochondria and restore the bioenergetics status of the cell. Hence, targeting pharmacological modulation of mitophagy might be a useful and promising strategy to counteract age-associated neurodegenerative disorders. Hence, several chemical and natural modulators have been investigated to maintain mitochondrial homeostasis and improve organismal healthspan.

### Iron chelators

There are three major iron consumption pathways such as iron storage, heme and iron sulfur cluster biosynthesis involved in proper function of mitochondria and maintenance of cellular iron homeostasis (Chen and Paw, 2012). Previous studies demonstrated mitophagy stimulation in different model organisms such as yeast, worms and mice upon iron depletion (Schiavi et al., 2015; Nagi et al., 2016; McWilliams et al., 2018; Palikaras et al., 2018a,b). It has been reported that the iron chelator deferiprone (DFP) induces mitochondrial elimination without altering mitochondrial membrane potential. The activation of Parkin is not required in DFP-mediated mitophagy suggesting that DFP might be used to enhance mitochondrial turnover in cells with defective PINK1/Parkin mechanism (Kondapalli et al., 2012; Allen et al., 2013). The siderophore-like chemical agents such as ciclopriox olamine and 1,10′-phenanthroline (Phen) promote mitophagy induction by triggering DRP-1 dependent mitochondrial fragmentation and dissipation of mitochondrial membrane (Kirienko et al., 2015; Palikaras et al., 2018a,b; Park et al., 2018). Interestingly, another siderophore 2′2-bipyridyl (BP) has been reported to enhance mitochondrial degradation through a PINK1, PDR-1, and DCT-1 dependent mechanism by triggering a hypoxia-like response in *C. elegans* (Schiavi et al., 2015). Additionally, BP treatment has also been shown to promote longevity through the mitophagy induction mechanism in *C. elegans* (Kropp et al., 2021). Several studies have demonstrated mitophagy-inducing properties of these iron chelators, further investigations are required to understand their underlying mechanisms of actions and therapeutic potential.

### Mitochondrial toxicants

The most commonly used mitochondrial toxicants as mitophagy inducers are proton ionophores which can cross the inner membrane of mitochondria and affect mitochondrial metabolism. These chemical compounds impair the balance between components of the electron transport chain (ETC) and oxidative phosphorylation by disrupting the electrochemical proton gradient. Mitochondrial uncouplers have been widely used in mammalian cells to influence mitochondrial degradation leading to elucidation of factors involved in the mitophagy process (Narendra et al., 2008; Palikaras et al., 2015). The most extensively used mitochondrial uncouplers including carbonyl cyanide m-chlorophenyl hydrazone (CCCP) and carbonyl cyanide-p-(trifluoromethoxy) phenylhydrazone (FCCP), which stimulate mitochondrial elimination by activating PINK-1/Parkin mediated mitophagy pathway (Narendra et al., 2010; Gatliff et al., 2014).

There are some limitations to use proton ionophores in biological research because of several undesirable properties of these agents. Their activity affects bioenergetics status of the cell, dissipation of mitochondrial membrane potential, lysosomal function, cytoskeleton homeostasis and ion channel stimulation (Wang et al., 2016; Georgakopoulos et al., 2017). Hence, unwanted activity of these agents interfere with mitochondrial metabolism and limit their therapeutics applications. A recent study has investigated a novel mitochondrial uncoupling agent called BAM15 which increases mitochondrial respiration rate (Kenwood and Brookside, 2014). Interestingly, BAM15 supplementation has been shown to reduce cytotoxicity and provide resistance to acute renal ischemic injury in mice, indicating its therapeutic potential against mitochondrial dysfunction. Consistent with these findings, the role of BAM15 in mitophagy induction has not yet been explored and further studies are required in this direction.

Additionally, several oxidative stress inducers such as paraquat, rotenone, 6-OHDA (6-hydroxyldoamine), and MPTP (1-methyl-4-phenyl-1,2,3,6-tetrahydropyridine) have been demonstrated as potent mitophagy mediators (Chu et al., 2013; Redmann et al., 2014; Palikaras et al., 2015; Georgakopoulos et al., 2017). These chemicals stimulate the generation of excessive ROS inducing mitochondrial damage and their subsequent degradation through mitophagy. 6-OHDA and MPTP treatments promote phosphorylation and recruitment of extracellular-signal regulated kinase 2 (ERK2) on mitochondrial surface which further facilitates sequestration of autophogosomes and removal of dysfunctional organelles. Moreover, these stress inducers promote mitochondrial removal *via* different molecular mechanisms but they share a common mitophagic response. The stress inducer paraquat activates increased generation of complex-I dependent superoxide which promotes mitochondrial depolarization and PINK1/Parkin pathway activation (Narendra et al., 2010; Palikaras et al., 2015). In contrast, rotenone or 6-OHDA supplemented neuronal cells cannot activate PINK1-mediated mitophagy due to their low effects on mitochondrial membrane potential. However, rotenone and 6-OHDA stimulate the release of cardiolipin from the intermembrane space of mitochondria which recruits autophagic machinery to the mitochondria facilitated by the direct association of cardiolipin and LC3.

### Natural bioactive molecules with mitophagy modulating properties

**Figure 3:**
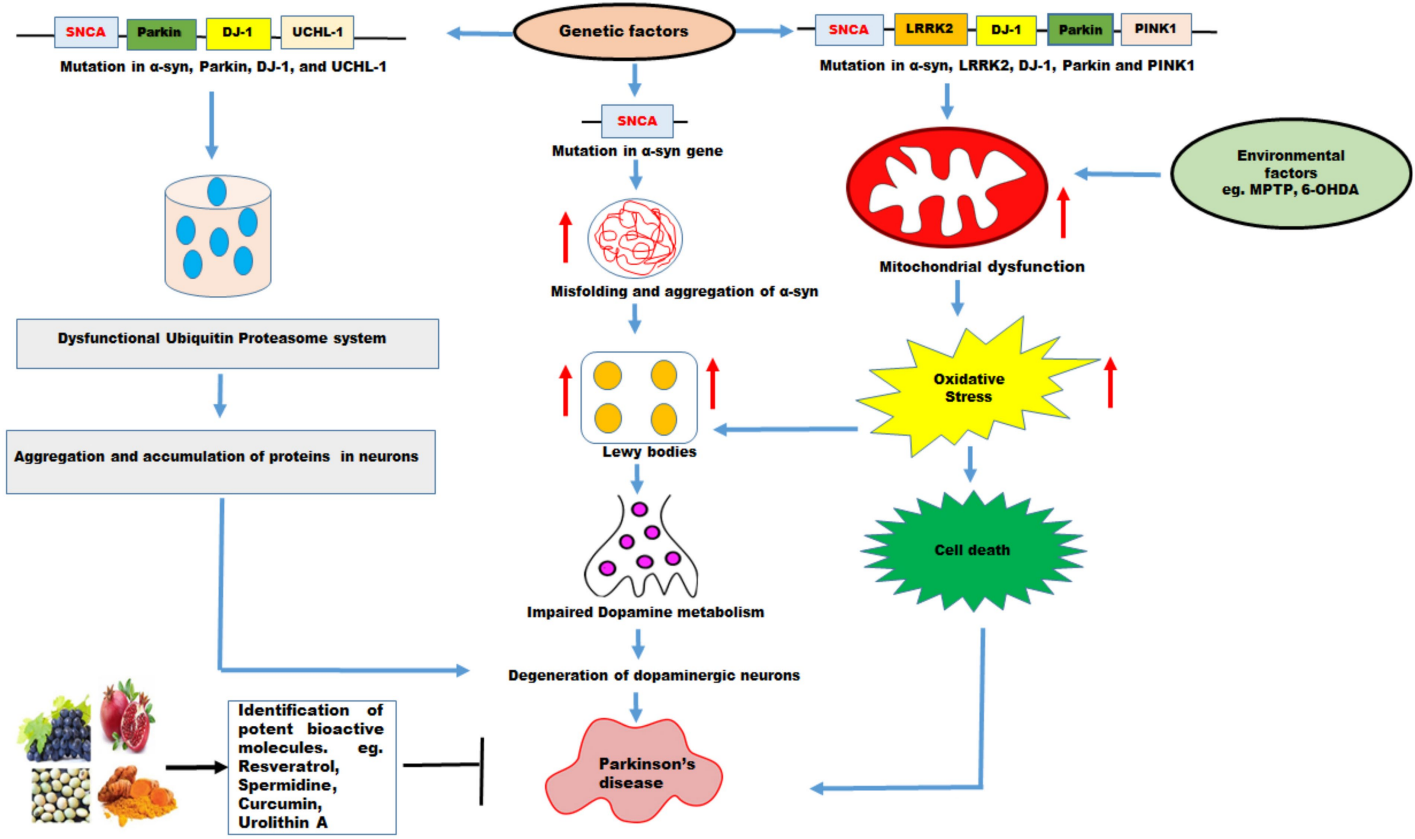
The effects of genetic and environmental factors on Parkinson’s disease. Genetic mutations in genes such as *SNCA, Parkin, DJ-1, UCHL-1, LRRK2, PINK1*, and environmental factors lead to PD. Mutations in α-syn cause misfolding and increases the aggregation of α-syn which further increase formation of Lewy bodies, impaired dopamine metabolism, and degeneration of DA neurons resulting in PD. Mutations in *α-syn*, *Parkin*, *DJ-1*, and *UCHL-1* impair ubiquitin proteasome system which lead to aggregation and accumulation of proteins in the neurons resulting in degeneration of DA neurons leading to PD. PD pathology has been associated with disruption in mitochondrial dynamics which exhibit mitochondrial dysfunction, increase oxidative stress leading to cell death. Mutations in *α-syn, LRRK2, DJ-1, Parkin* and *PINK1* and environmental factors such as MPTP, and 6-OHDA promote mitochondrial dysfunction and increased oxidative stress leading to cell death. The natural molecules possess variety of health promoting benefits and these molecules function as mitophagy inducers which pharmacologically modulate the mitophagy and might be useful in PD therapy.

The role of natural bioactive molecules has gained considerable interest as alternative options for Parkinson’s disease treatment due to the lack of success of PD-targeted approaches in pharmacological research (Figure 3). Polyphenols have been reported for delaying aging and age-related neurodegenerative diseases such as PD due to their versatile health benefits (Bhullar and Rupasinghe, 2013). The use of these polyphenols for therapy is a major challenge in pharmaceutical industry because of their poor bioavailability after ingestion and incapability to cross the blood brain barrier. The most extensively studied polyphenols that might be useful for PD prevention includes resveratrol, curcumin, catechin, quercetin, and recently used urolithin A (Table 2). These molecules may also catalyze mitochondrial turnover and biogenesis.

**Table 2:** List of natural molecules with their mechanism of action.

Natural molecules	Major source	Mechanism of actions	References
Resveratrol	Grapes	Anti-aging, anti-cancerous, Mitophagy	Bass et al., 2007; Wu et al., 2011; Wu et al., 2014
Urolithin A	Pomegranate	Mitophagy, longevity	Ryu et al., 2016
Curcumin	*Curcuma longa* root	Anti-cancerous, mitophagy	Bimonte et al., 2015; de Oliveira et al., 2016
Quercetin	Apple, strawberries	stress resistance, longevity, mitophagy	Kampkötter et al., 2008; Wang et al., 2021
Catechin	*Camellia sinensis*	Mitophagy, longevity	Wu et al., 2020
Spermidine	Mushrooms, soy products	Autophagy	Ghosh et al., 2020
Metformin	*Galega officinalis*	Anti-aging, mitophagy	Palikaras et al., 2018a,b; Hu et al., 2021
Fisetin	Strawberries, Apples	Autophagy	Jia et al., 2019
Tomatidine	Tomato leaves	Stress resistance, longevity	Dilberger et al., 2019
Kaempferol Trehalose	Green leafy vegetables Bacteria, yeast, fungi, insects, invertebrates, and plants.	Stress resistance, longevity Anti-oxidant, anti-inflammatory, neuroprotective effects, autophagy inducer.	Kampkötter et al., 2007 Hosseinpour-Moghaddam et al., 2018; Khalifeh et al., 2021

### Resveratrol: A wonder molecule

Resveratrol (3,4,5-trihydroxy-trans-stilbene) is a natural polyphenolic compound found in common foods such as berries, peanuts, red wine and red grapes skin (Bhat et al., 2001). Polyphenols are compound which exhibit neuroprotective effects through several mechanisms including ROS scavenging, inhibiting oxidant enzymes, inducing autophagy, modulating mitochondrial transduction pathway and mitochondrial dynamics (Bhullar and Rupasinghe, 2013; Pallauf and Rimbach, 2013; Naoi et al., 2019). Among the polyphenols, resveratrol displays potent neuroprotection efficacy in several experimental models of AD and PD. However, its clinical application is limited due to low bioavailability and rapid metabolism. One study demonstrated that when SH-SY5Y cells were supplemented with both rotenone (a mitochondrial complex I inhibitor) and resveratrol simultaneously, resveratrol prevented rotenone induced apoptosis by inducing autophagy and increasing overall autophagy flux thereby exhibiting neuroprotective effect (Lin et al., 2014). Furthermore, resveratrol also influenced mitochondrial homeostasis by inducing mitochondrial biogenesis and energy metabolism *via* AMPK pathway (Wu et al., 2011). Reports show that resveratrol treatment also increases the expression of mitofusin 2 and known mitochondrial biogenesis regulator PGC-1α, which restores muscular functions and healthy mitochondrial morphology (Lagouge et al., 2006). Additionally, MPTP or rotenone induced mitochondrial dysfunction in cells treated with resveratrol exhibit reduced level of cytochrome c and activated caspase 3, suggesting reduced apoptosis in the cells. Reduced apoptosis slows the progression of PD. Resveratrol pre-treatment induces autophagy (mitophagy) promoting longevity and organismal healthspan in SIRT1-dependent manner, suggesting that mitochondrial elimination is a result of general autophagy induction instead of a selective cellular response (Bass et al., 2007). In a study, it was observed that resveratrol provides neuroprotection in response to H_2_O_2_/6-OHDA by activating SIRT1 (Kao et al., 2010). Interestingly, resveratrol ameliorates the toxicity of α-synuclein aggregation through SIRT-1 activation, however, the underlying mechanism is not fully explained (Sun et al., 2010; Ganguly et al., 2018). Recent study has indicated that SIRT1 stimulates basal autophagy while accumulation of defective organelles and disruption in energy homeostasis have been observed in the absence of SIRT1, suggesting that SIRT1 plays key role in the autophagy regulation. Taken together, resveratrol mediated mitophagy induction and mitochondrial biogenesis might be useful for preventing DA neurons death in PD patients. However, the underlying mechanism for resveratrol mediated neuroprotection against PD remains to be fully explored.

## Curcumin

The polyphenol curcumin is a main component of turmeric (*Curcuma longa*) and derived from its rhizome. In traditional Indian Ayurvedic medicine systems, turmeric compounds like curcumin have been used for the treatment of various ailments including gastrointestinal and pulmonary diseases (Ganguly et al., 2018). Curcumin possesses versatile biological properties such as antioxidant, anti-cancerous, anti-inflammatory and neuroprotective effects (Hewlings and Kalman, 2017). Curcumin also acts like a tumor suppressor and provide protection against cancerous cell proliferation (Shanmugam et al., 2015). Curcumin produces inhibitory effects on growth of breast cancer cells and metastasis in mice bearing breast cancer (Bimonte et al., 2015). Curcumin mediated autophagy induction upregulates LC3-II and Beclin 1 which inhibits *in vitro* cell proliferation of chronic granulocyte leukemia, oesophageal and glioblastoma cancer (Lee et al., 2019; Shakeri et al., 2019).

Consistent with these studies, curcumin has been demonstrated to induce PINK1/Parkin mediated mitophagy by inhibiting the Akt/mTOR pathway. Furthermore, curcumin enhanced the mitochondrial biogenesis by modulating expression of genes such as *SIRT1* and *PGC-1α* (de Oliveira et al., 2016). The curcumin-mediated mitophagy induction was also found in malignant glioma cells. Liao et al. investigated the effects of curcumin on aging and stress parameters using the *C. elegans* model system and they proposed curcumin mediated lifespan extension and attenuation of ROS level *(*Liao et al., 2011). Similarly, Wang and Xu, also demonstrated that curcumin can improve healthspan by inducing mitophagy and restoring the mitochondrial function in neurons of rats (Wang and Xu, 2020). Therefore, several approaches have been explored to increase the solubility and absorption of curcumin, but its oral use for humans is still under debate due to its low bioavailability. Moreover, curcumin (10 μM) was also found to sensitize the CNE2 cells (nasopharyngeal carcinoma) before being exposed to ultrasound. The combination of ultrasound and curcumin induce swelling of mitochondria lead to their dysfunction (Momtazi-Borojeni et al., 2018). These findings suggest that a specific autophagy mechanism can eliminate damaged mitochondria in response to curcumin. However, additional data are required to evaluate the role of curcumin in mitophagy induction in more detail.

## Spermidine

Spermidine is a polyamine synthesized from putrescine and performs like a precursor molecule of spermine generation. Polyamines are small molecules which impact cellular viability by modulating different processes including cell growth, proliferation, innate immune responses and energy homeostasis (Miller-Fleming et al., 2015). During aging, the cellular level of spermidine is found reduced in various tissues like heart, kidney, liver and thymus among other tissues. Therefore, metabolism of polyamine and associated pathways are directly correlated with physiology of organisms. Previous studies highlighted that polyamine levels gradually decline with aging and develop pathological conditions in organisms (Markaki et al., 2018; Palikaras et al., 2018a,b; Ni and Liu, 2021). The decreased intracellular level of spermidine may disrupt cellular homeostasis. Spermidine mediated longevity promotion and amelioration of oxidative stress have been reported in several model organisms such as yeast, worms, flies and mice through autophagy (Eisenberg et al., 2009; Madeo et al., 2010; Schwarz et al., 2018; Maruzs et al., 2019; Yang et al., 2020). Interestingly, spermidine also activates autophagy and mitophagy in cardiac muscles, reduces the level of TNF (pro-inflammatory cytokine) and enhances mitochondrial metabolism (Tong and Hill, 2017). Spermidine has been demonstrated to induce mitophagy by upregulating the expression level of mitophagy markers such as Atgs, Beclin-1, PINK1, Parkin, LC3-II, and ULK1 and through mTOR inhibition in animal and cellular models (Palikaras et al., 2018a,b; Yang et al., 2020). Mice fed with spermidine supplement upregulated mitophagy in aged neurons as well as in case of neuroblastoma. Ghosh et al., proposed that spermidine activates autophagy and reduces the ROS formation to counteract age-related cell death (Ghosh et al., 2020).

Qi *et al* have demonstrated that ATM kinase activate PINK1/Parkin pathway facilitating mitochondrial removal in response to spermidine in human fibroblasts. Spermidine promotes depletion mitochondrial membrane potential leading to activation of ATM kinase and recruits Parkin on mitochondrial surface. In contrast, ATM inhibitors were found to prevent recruitment of Parkin, reduce the expression of PINK1, and suppress mitophagy in spermidine supplemented mammalian cells (Qi et al., 2016). Therefore, several lines of evidences suggest that dietary administration of spermidine might be useful for healthy aging and providing protection against neurodegenerative disorders in elderly population through targeting autophagy and mitophagy (Eisenberg et al., 2016).

### Urolithin A: Mitophagy inducer

Urolithins are 6H-dibenzopyran-6-one derivatives (or aglycons) produced by gut microbiota. Ellagitannins are the main precursor of urolithin that occur in fruits such as pomegranate, blackberries, raspberries and strawberries and walnuts. Urolithins have been recognized as emerging therapeutic agents due to their health benefits against diseases, including cardiac dysfunction, inflammation and carcinogenesis (D’Amico et al., 2021). In previous study, Ryu et al., demonstrated the significant role of urolithins in cellular homeostasis and longevity promotion. The pomegranate fruit is the richest source of ellagitannins (ETs) for humans, whereas ETs are hydrolysed in the gut to produce ellagic acid (EA) which is further processed by gut microflora into urolithins (Ryu et al., 2016). Urolithin A (UA) is the most abundant metabolite among other urolithins (UB, UC and UD) that is considered to be the end product of both ETs and EA in species including humans investigated till date. Remarkably, it was found that UA promotes longevity by specifically inducing mitophagy in nematodes and rodents (Ryu et al., 2016). Similarly, UA supplementation reduces decline in pharyngeal pumping, age-dependent mobility defect and improves energy metabolism in *C. elegans*. Ryu et al, observed that UA treatment induces the formation of LGG-1-GFP (LC3-GFP in mammals) positive punctae in *C. elegans* (Ryu et al., 2016). Mitochondrial elimination was completely suppressed in UA exposed worms following knockdown of *Beclin-1* (*bec-1*), main regulator of autophagy or the autophagosome adaptor sequestosome/p62 (*sqst-1*). Similar effect was observed like *bec-1* and *sqst-1* in worms after silencing the mitophagy using RNAi against genes such as *pink1* (PTEN induced kinase-1) and BNIP3 (BCL2/adenovirus E1B interacting protein 3) in *C. elegans* (Ryu et al., 2016). Similarly, a dose dependent induction of autophagy (the ratio of LC3-II/LC3-I in the total cell lysate) and mitophagy (ubiquitination and p62/SQSTM in the mitochondrial fraction) was observed in UA treated mammalian cells C2C12 myoblast and Mode-K intestinal cells (D’Amico et al., 2021). UA has also been exhibited a prominent role in mitophagy induction and improvement in muscle function in rodent models (Luan et al., 2021). Mitophagy regulators such as PINK1, Beclin 1, p62/SQSTM1 and BNIP3/NX were found to be involve in UA-mediated longevity promotion, suggesting that UA supplementation maintains mitochondrial function and organismal health span through several interconnected regulatory mechanisms of mitophagy (Palikaras et al., 2018a,b). UA also enhances mitochondrial biogenesis by activating SKN-1 to maintain mitochondrial activity and promote SKN-1 mediated lifespan extension.

UA has been demonstrated as non-genotoxic molecule with LD50 value (lethal dose 50) greater than 5 g/kg body weight in rats (Patel et al., 2008). Furthermore, clinical trials concluded that UA is a safe and bioavailable in humans and is effective against age-associated neurodegeneration. Mitophagy induction and mitochondrial biogenesis are crucial mechanism to maintain the healthy population of mitochondria. The activation of AMPK, SIRT1, PGC1α and inhibition of mTOR pathway have also been shown to induce mitophagy (Palikaras et al., 2017; Jayatunga et al., 2021). UA mediated modulations of these key signalling pathways have been reported in previous studies (Ryu et al., 2016). It has been shown that SIRT deacetylates and activates an upstream kinase, LKB1 which activates AMPK (Pillai et al., 2010). The levels of ATP/NAD+ has also found to be increased by UA which activates SIRT1 promoters and affect SIRT1/PGC1 α pathway (Ghosh et al., 2020). UA has also been shown to increase the mitofusin 2 (*mfn2*) expression levels in its mitophagy inducing pathway (Sebastián et al., 2016; Andreux et al., 2019). Previous studies proposed that UA enhances *Parkin* and *BECN1* transcriptional levels in humans after 28 days of UA supplementation (Andreux et al., 2019). Many cell signaling pathways such as induction of downstream mitophagy effectors ULK1 by inhibiting mTOR involves AMPK activation. UA mediated activation of AMPK and modulates mTOR pathway has been previously reported (Zhang et al., 2021). Based on the previous findings, UA may induce ULK1 activation through AMPK which may further phosphorylate LIR motif of FUNDC1 to induce mitophagy (Andreux et al., 2019). Therefore, these findings highlight that UA can reinstate metabolic energy by inducing mitophagy mechanism which can be further utilized as a novel therapeutic intervention to improve health span and prevent age-associated diseases like PD in the elderly.

Therefore, the present study summarised the completed clinical trials of natural molecules used as mitophagy inducers in Parkinson’s disease (Table 3). This clinical trial data has been collected from different literature, which have been presented an overview of clinical trials study using treatments (curcumin, resveratrol, spermidine and urolithin A) at different doses and duration to observe the outcome of this study (Wirth et al., 2018; Singh et al., 2019; Ghodsi et al., 2022; Liu et al., 2022).

**Table 3:** Summary of clinical trials in Parkinson’s disease using mitophagy inducers.

Treatment	Target	Type of study	Dose	Duration	Study	Year	Outcome
Curcumin	PD patients aged ≥30 ≥ 30 for curcumin and placebo group	Randomized, triple-blind, placebo-controlled	80 mg/Day	9 months	Ghodsi et al.,	2022	The movement disorder was observed in primary outcome. This clinical trial was not successful in showing clinical symptoms of PD.
Resveratrol	AD patients (n = 119)	Phase II trial completed	500 mg/day, 2 × 2000 mg/day, 52 weeks	23 months	Singh et al.,	2019	The pathological changes were observed in patient’s brain. The analysis suggested that resveratrol can cross the BBB and neuroprotection was not demonstrated.
	AD patients (*n* = 27)	Phase III trial completed	Dietary	42 months	Singh et al.,	2019	Improved memory performance.
	PD (*n* = 40)	Phase I trial	6 × 25, 50, 100 and 150 mg/d, 2d	3 months	Singh et al.,	2019	
	PD (*n* = 25)	Phase I trial	200 mg/d, 1^st^ day, 3 × 200 mg/d, 2^nd^ and 3^rd^ day, 200 mg/d, 4^th^ day	3 months	Singh et al.,	2019	
	PD (*n* = 20)	Phase I trial	25, 50,100 and 200 mg/d, 5 × 1 d	3 months	Singh et al.,	2019	
	PD (*n* = 24)	Phase I trial	400 mg/d, 1 d	3 months	Singh et al.,	2019	
	PD (*n* = 38)	Phase I trial	3 × 25, 50, 75 and 100 mg/d, 1^st^-4^th^ day; 1 × 25–100 mg/d, 5^th^ day	4 months	Singh et al.,	2019	
	PD (*n* = 39)	Phase I trial	3 × 25, 50, 75 and 100 mg/d, 1^st^-4^th^ day; 1 × 25–100 mg/d, 5^th^ day	4 months	Singh et al.,	2019	
Spermidine	AD patients (*n* = 30, 60–80 years of age)	Randomized, placebo-controlled, double-blind Phase IIa pilot trial	3capsules/day, Polyamine supplements	3 months	Wirth et al.,	2018	Changes in cognitive function. Lower the risk of cognitive impairment in humans.
	PD	-------	-------	-------	-------	------	No clinical trial studies have been reported till now.
Urolithin A	Older adults (*n* = 66, 65–90 years of age)	Randomized, placebo-controlled, double-blind, clinical trial	1,000 mg	2–4 months	Liu et al.,	2022	Urolithin A is a safe drug that benefits mitochondrial health and muscle endurance in older adults. This finding might be useful to treat age-related diseases.

PD-Parkinson’s disease, AD-Alzheimer’s disease.

### The antibiotics as mitophagy enhancer

Several studies have been found that antibiotics modulate mitophagy at certain concentrations while overuse of certain antibiotics can cause mitochondrial dysfunction, disruption of mitochondrial homeostasis, failure in energy metabolism and development of diseases (Palikaras et al., 2018a,b). An inhibitor of mitochondrial complex III antimycin A, stimulates overproduction of ROS resulting in mild depolarization of membrane but antimycin A treatment activates F_1_F_0_-ATP synthase which can re-establish membrane potential (Georgakopoulos et al., 2017). Consequently, antimycin A is regularly used in combination with F_1_F_0_-ATP synthase inhibitor oligomycin to augment mitochondrial defects and thereby induce PINK1-Parkin mediated mitophagy pathway (Lazarou et al., 2015). Furthermore, other antibiotics such as salinomycin and valinomycin disrupt potassium homeostasis in mitochondria which leads to their dysfunction and activation of PINK1-Parkin mediated mitophagy. Accumulating evidence suggests that an antibacterial compound actinonin triggers mitochondrial removal resulting in organelle proteotoxicity (Richter et al., 2013). Interestingly, stabilized PINK1 phosphorylates Parkin on mitochondrial membrane, where it interacts with autophagosomal protein LC3 to facilitate mitochondrial degradation upon actinonin administration (Burman et al., 2017). Previous studies have demonstrated that tetracycline effects morphology of mitochondria, proteostasis and nuclear gene expression in *Drosophila*, *C. elegans* and mice (Moullan et al., 2015). Thus, the use of antibiotics as potent mitophagy inducer in controlled manner can provide valid means to develop novel therapeutics strategies against neurodegenerative disorders for future prospects.

### Mitophagy inhibition

Mitophagy inhibition may be necessary in situation where excess mitophagy needs to be controlled. The uncontrolled removal of mitochondria leads to reduction of mitochondrial population this can induce stress in other organelles resulting in activation of apoptosis. Several chemical or natural molecules have been proposed for pharmacological inhibition of mitophagy (Georgakopoulos et al., 2017). For instance, 3-methyladenine, an autophagy inhibitor is commonly utilized for mitophagy suppression (Wu et al., 2010). The lysosomal inhibitors such as bafilomycin and chloroquine disrupt fusion of autophagosome to lysosome membrane by preventing lysosomal acidification (Redmann et al., 2017). Thus, these lysosomal inhibitors play major role in prevention of mitochondrial degradation. However, due to non-selective function of these compounds, an alternative method like modulation in mitochondrial fission is required for preventing mitochondrial elimination. In yeast and mammalian cells, mutations in *mitochondrial division inhibitor 1* (*mdivi-1*) disrupt morphology of mitochondria by preventing its division. Remarkably, mdivi-1 impairs the enzymatic activity of Drp1 which reduces cell death and apoptosis (Bordt et al., 2017). Recently, peptide inhibitors are recognized as new approach to modulate selective mitophagy pathway. However, additional research is required to investigate mimetic peptides for mitophagy modulation.

### Mitophagy modulation as therapeutic approach in Parkinson’s pathogenesis

The impairment in mitophagy is a common denominator of age-related neurodegenerative diseases. Therefore, pharmacological interventions with potent chemical or natural modulators targeting mitophagy might have therapeutic potential for treating various ailments. Several studies have identified chemical and natural compounds to restore the effective removal of damaged organelles by manipulating the mitophagy mechanism (Georgakopoulos et al., 2017). Metformin and rapamycin are commonly used autophagy inducing drugs which preserve energy metabolism through mitophagy induction by attenuating mTOR and AMPK activation (Shi et al., 2012; Villanueva Paz et al., 2016). Metformin supplementation induces mitophagy by stimulating Parkin activation through downregulation of p53. The suppression of mitochondrial elimination might occur by direct interaction of cytoplasmic p53 with Parkin (Palikaras et al., 2018a,b). Additionally, rapamycin administration exhibit protective effects against impaired mitochondrial function by maintaining stress resistance and energy homeostasis (Li et al., 2014). Several natural compounds such as resveratrol, urolithin A, spermidine, and certain antibiotics have been identified as mitophagy inducers which maintain mitochondrial integrity, and promote anti-aging effects in different model organisms including yeast, flies, nematodes and mice (Palikaras et al., 2018a,b). However, further investigation is required to uncover their underlying mechanism of action. The development of therapeutics interventions by using compounds having biogenic and mitophagic activities may affect human health span physiology and age-associated neurodegenerative disorders. Furthermore, it has also been demonstrated that Vitamin K2 plays an important role in electron transfer in mitochondria of Drosophila (Lin et al., 2021). This vitamin may function like electron carrier and induces ATP generation in mitochondria. Vitamin K2 helps in maintaining normal ATP production which may provide new approach for PD treatment (Vos et al., 2012). Therefore, co-ordination between mitochondrial biogenesis and degradation is much needed for maintaining mitochondrial functions and homeostasis in organism. The identification of potent chemical/natural mitophagy modulators could be a promising interventions for PD treatment by targeting mitophagy manipulation.

### Concluding remarks and future prospects

This review illustrated the exploration of the different mitophagy pathways, mitophagy modulators and their therapeutics approach in PD prevention with main focus on utilizing *Drosophila* as a model. In this context, several studies have targeted mitophagy modulation as a potential therapy for PD. Several risk factors and PD-related genes are associated with mitophagy impairment. Mitochondria and autophagy are strongly interconnected and their impairment contributes to PD pathogenesis. However, mitophagy modulation is considered as promising approach in PD prevention, but mitophagy regulation and its underlying mechanism in PD pathogenies is still unclear. Many open questions are still persisting: what are the specific mechanisms underlying lysosomal and mitochondrial dysfunction and how their interaction leads to PD pathogenesis? What is the role of mitophagy modulation in mitochondrial quality control in neurons as compared to other mitochondrial degradation pathways? How mitophagy and mitochondrial biogenesis are connected with each other, how these are associated to mitochondrial fission and fusion process in neurons? Despite many debates in this field, we still need to address these questions by targeting mitophagy and restoring energy metabolism which may provide a therapeutic approach for PD pathophysiology. The purpose of these studies is to identify potential drug candidates for mitophagy modulation and decipher their mode of action. The knowledge generated from such studies can hopefully be used to conduct further research in higher organisms potentially leading to clinical trials.

## Author contributions

JA wrote the entire manuscript, tabulated the information, and designed the figures. JA and BS edited the all versions of the manuscript. All authors contributed to the article and approved the submitted version.

## Funding

This work was supported by grants received from DST-Science and Engineering Research Board (Grant number PDF/2020/000684).

## Conflict of interest

The authors declare that the research was conducted in the absence of any commercial or financial relationships that could be construed as a potential conflict of interest.

## Publisher’s note

All claims expressed in this article are solely those of the authors and do not necessarily represent those of their affiliated organizations, or those of the publisher, the editors and the reviewers. Any product that may be evaluated in this article, or claim that may be made by its manufacturer, is not guaranteed or endorsed by the publisher.

## References

[ref1] Abolaji A. O. Adedara A. O. Adie M. A. Vicente-Crespo M. Farombi E. O. (2018). Resveratrol prolongs lifespan and improves 1-methyl-4-phenyl-1,2,3,6-tetrahydropyridine-induced oxidative damage and behavioural deficits in Drosophila melanogaster. Biochem. Biophys. Res. Commun. 503, 1042–1048. doi: 10.1016/j.bbrc.2018.06.114, PMID: 29935183

[ref2] Abou-Sleiman P. M. Muqit M. M. Wood N. W. (2006). Expanding insights of mitochondrial dysfunction in Parkinson's disease. Nat. Rev. Neurosci. 7, 207–219. doi: 10.1038/nrn1868, PMID: 16495942

[ref3] Allen G. F.. (2013). Loss of iron triggers PINK1/Parkin-independent mitophagy. EMBO Rep. 14, 1127–1135. doi: 10.1038/embor.2013.168, PMID: 24176932 PMC3981094

[ref4] Andreux P. A. Blanco-Bose W. Ryu D. Burdet F. Ibberson M. Aebischer P.. (2019). The mitophagy activator urolithin A is safe and induces a molecular signature of improved mitochondrial and cellular health in humans. Nat. Metab. 1, 595–603. doi: 10.1038/s42255-019-0073-4, PMID: 32694802

[ref5] Aryal B. Lee Y. (2019). Disease model organism for Parkinson disease: *Drosophila melanogaster*. BMB Rep. 52, 250–258. doi: 10.5483/BMBRep.2019.52.4.204, PMID: 30545438 PMC6507844

[ref6] Auluck P. K. Bonini N. M. (2002). Pharmacological prevention of Parkinson disease in drosophila. Nat. Med. 8, 1185–1186. doi: 10.1038/nm1102-1185, PMID: 12411925

[ref7] Auluck P. K. Chan H. Y. E. Trojanowski J. Q. Lee V. M. Y. Bonini N. M. (2002). Chaperone suppression of α-synuclein toxicity in a *Drosophila* model for Parkinson's disease. Science 295, 865–868. doi: 10.1126/science.1067389, PMID: 11823645

[ref8] Bass T. M. Weinkove D. Houthoofd K. Gems D. Partridge L. (2007). Effects of resveratrol on lifespan in Drosophila melanogaster and Caenorhabditis elegans. Mech. Ageing Dev. 128, 546–552. doi: 10.1016/j.mad.2007.07.007, PMID: 17875315

[ref9] Behl T. Kaur G. Fratila O. Buhas C. Judea-Pusta C. T. Negrut N.. (2021). Cross-talks among GBA mutations, glucocerebrosidase, and α-synuclein in GBA-associated Parkinson’s disease and their targeted therapeutic approaches: a comprehensive review. Transl. Neurodegener. 10, 1–13. doi: 10.1186/s40035-020-00226-x 33446243 PMC7809876

[ref10] Betarbet R. Sherer T. B. Greenamyre J. T. (2002). Animal models of Parkinson's disease. BioEssays 24, 308–318. doi: 10.1002/bies.10067 11948617

[ref11] Bhat K. P. Kosmeder J. W. Pezzuto J. M. (2001). Biological effects of resveratrol. Antioxid. Redox Signal. 3, 1041–1064. doi: 10.1089/152308601317203567 11813979

[ref12] Bhujabal Z. Birgisdottir Å. B. Sjøttem E. Brenne H. B. Øvervatn A. Habisov S.. (2017). FKBP8 recruits LC3A to mediate Parkin-independent mitophagy. EMBO Rep. 18, 947–961. doi: 10.15252/embr.201643147, PMID: 28381481 PMC5452039

[ref13] Bhullar K. S. Rupasinghe H. (2013). Polyphenols: multipotent therapeutic agents in neurodegenerative diseases. Oxidative Med. Cell. Longev. 2013, 1–18. doi: 10.1155/2013/891748, PMID: 23840922 PMC3690243

[ref14] Bimonte S. Barbieri A. Palma G. Rea D. Luciano A. D’Aiuto M.. (2015). Dissecting the role of curcumin in tumour growth and angiogenesis in mouse model of human breast cancer. Biomed. Res. Int. 2015, 1–7. doi: 10.1155/2015/878134, PMID: 25879038 PMC4386568

[ref15] Blandini F. Armentero M. T. (2012). Animal models of Parkinson’s disease. FEBS J. 279, 1156–1166. doi: 10.1111/j.1742-4658.2012.08491.x 22251459

[ref16] Bolus H. Crocker K. Boekhoff-Falk G. Chtarbanova S. (2020). Modeling neurodegenerative disorders in *Drosophila melanogaster*. Int. J. Mol. Sci. 21:3055. doi: 10.3390/ijms21093055, PMID: 32357532 PMC7246467

[ref17] Bonello F. Hassoun S. M. Mouton-Liger F. Shin Y. S. Muscat A. Tesson C.. (2019). LRRK2 impairs PINK1/Parkin-dependent mitophagy via its kinase activity: pathologic insights into Parkinson’s disease. Hum. Mol. Genet. 28, 1645–1660. doi: 10.1093/hmg/ddz004, PMID: 30629163

[ref18] Bonifati V. Rizzu P. Squitieri F. Krieger E. Vanacore N. van Swieten J. C.. (2003). DJ-1 (PARK7), a novel gene for autosomal recessive, early onset parkinsonism. Neurol. Sci. 24, 159–160. doi: 10.1007/s10072-003-0108-0, PMID: 14598065

[ref19] Bordt E.A. Clerc P. Roelofs B.A. Saladino A.J. Tretter L. Adam-Vizi V. Cherok E. Khalil A. Yadava N. Ge S.X. Francis T.C. Kennedy N.W. Picton L.K. Kumar T. Uppuluri S. Miller A.M. Itoh K. Karbowski M. Sesaki H. Hill R.B. Polster B.M., The putative Drp1 inhibitor mdivi-1 is a reversible mitochondrial complex I inhibitor that modulates reactive oxygen species. Dev. Cell, 2017. 40: p. 583–594. e6, 594.e6, doi: 10.1016/j.devcel.2017.02.020, PMID: . 28350990 PMC5398851

[ref20] Breda C. Nugent M. L. Estranero J. G. Kyriacou C. P. Outeiro T. F. Steinert J. R.. (2015). Rab11 modulates α-synuclein-mediated defects in synaptic transmission and behaviour. Hum. Mol. Genet. 24, 1077–1091. doi: 10.1093/hmg/ddu521, PMID: 25305083 PMC4986550

[ref21] Breger L. S. Fuzzati Armentero M. T. (2019). Genetically engineered animal models of Parkinson's disease: from worm to rodent. Eur. J. Neurosci. 49, 533–560. doi: 10.1111/ejn.14300, PMID: 30552719

[ref22] Brockmann K. Srulijes K. Pflederer S. Hauser A.-K. Schulte C. Maetzler W.. (2015). GBA-associated Parkinson's disease: reduced survival and more rapid progression in a prospective longitudinal study. Mov. Disord. 30, 407–411. doi: 10.1002/mds.26071, PMID: 25448271

[ref23] Burman J. L. Pickles S. Wang C. Sekine S. Vargas J. N. S. Zhang Z.. (2017). Mitochondrial fission facilitates the selective mitophagy of protein aggregates. J. Cell Biol. 216, 3231–3247. doi: 10.1083/jcb.201612106, PMID: 28893839 PMC5626535

[ref24] Cackovic J. Gutierrez-Luke S. Call G. B. Juba A. O’Brien S. Jun C. H.. (2018). Vulnerable parkin loss-of-function *Drosophila* dopaminergic neurons have advanced mitochondrial aging, mitochondrial network loss and transiently reduced autophagosome recruitment. Front. Cell. Neurosci. 12:39. doi: 10.3389/fncel.2018.00039, PMID: 29497364 PMC5818410

[ref25] Cha G.-H. Kim S. Park J. Lee E. Kim M. Lee S. B.. (2005). Parkin negatively regulates JNK pathway in the dopaminergic neurons of *Drosophila*. Proc. Natl. Acad. Sci. 102, 10345–10350. doi: 10.1073/pnas.0500346102, PMID: 16002472 PMC1177361

[ref26] Chen C. Paw B. H. (2012). Cellular and mitochondrial iron homeostasis in vertebrates. Biochim. Biophys. Acta - Mol. Cell Res. 1823, 1459–1467. doi: 10.1016/j.bbamcr.2012.01.003 PMC3350831 22285816

[ref27] Chen J. Ren Y. Gui C. Zhao M. Wu X. Mao K.. (2018). Phosphorylation of Parkin at serine 131 by p38 MAPK promotes mitochondrial dysfunction and neuronal death in mutant A53T α-synuclein model of Parkinson’s disease. Cell Death Dis. 9, 1–15. doi: 10.1038/s41419-018-0722-7 29899409 PMC5999948

[ref28] Chen A. Y. Xia S. Wilburn P. Tully T. (2014). Olfactory deficits in an alpha-synuclein fly model of Parkinson’s disease. PLoS One 9:e97758. doi: 10.1371/journal.pone.0097758, PMID: 24879013 PMC4039441

[ref29] Chu C. T. (2019). Multiple pathways for mitophagy: a neurodegenerative conundrum for Parkinson’s disease. Neurosci. Lett. 697, 66–71. doi: 10.1016/j.neulet.2018.04.004, PMID: 29626647 PMC6170746

[ref30] Chu C. T. Bayır H. Kagan V. E. (2014). LC3 binds externalized cardiolipin on injured mitochondria to signal mitophagy in neurons: implications for Parkinson disease. Autophagy 10, 376–378. doi: 10.4161/auto.27191, PMID: 24351649 PMC5396091

[ref31] Chu C. T. Ji J. Dagda R. K. Jiang J. F. Tyurina Y. Y. Kapralov A. A.. (2013). Cardiolipin externalization to the outer mitochondrial membrane acts as an elimination signal for mitophagy in neuronal cells. Nat. Cell Biol. 15, 1197–1205. doi: 10.1038/ncb2837, PMID: 24036476 PMC3806088

[ref32] Clark I. E. Dodson M. W. Jiang C. Cao J. H. Huh J. R. Seol J. H.. (2006). Drosophila pink1 is required for mitochondrial function and interacts genetically with parkin. Nature 441, 1162–1166. doi: 10.1038/nature04779, PMID: 16672981

[ref33] Clark E. H. Vázquez de la Torre A. Hoshikawa T. Briston T. (2021). Targeting mitophagy in Parkinson's disease. J. Biol. Chem. 296:100209. doi: 10.1074/jbc.REV120.014294, PMID: 33372898 PMC7948953

[ref34] Cooper A. A. Gitler A. D. Cashikar A. Haynes C. M. Hill K. J. Bhullar B.. (2006). α-Synuclein blocks ER-Golgi traffic and Rab1 rescues neuron loss in Parkinson's models. Science 313, 324–328. doi: 10.1126/science.1129462, PMID: 16794039 PMC1983366

[ref35] D’Amico D. Andreux P. A. Valdés P. Singh A. Rinsch C. Auwerx J. (2021). Impact of the natural compound urolithin a on health, disease, and aging. Trends Mol. Med. 27, 687–699. doi: 10.1016/j.molmed.2021.04.009, PMID: 34030963

[ref36] Davis M. Y. Trinh K. Thomas R. E. Yu S. Germanos A. A. Whitley B. N.. (2016). Glucocerebrosidase deficiency in *Drosophila* results in α-synuclein-independent protein aggregation and neurodegeneration. PLoS Genet. 12:e1005944. doi: 10.1371/journal.pgen.1005944, PMID: 27019408 PMC4809718

[ref37] de Oliveira M. R. Jardim F. R. Setzer W. N. Nabavi S. M. Nabavi S. F. (2016). Curcumin, mitochondrial biogenesis, and mitophagy: exploring recent data and indicating future needs. Biotechnol. Adv. 34, 813–826. doi: 10.1016/j.biotechadv.2016.04.004, PMID: 27143655

[ref38] Devi L. Raghavendran V. Prabhu B. M. Avadhani N. G. Anandatheerthavarada H. K. (2008). Mitochondrial import and accumulation of α-synuclein impair complex I in human dopaminergic neuronal cultures and Parkinson disease brain. J. Biol. Chem. 283, 9089–9100. doi: 10.1074/jbc.M710012200, PMID: 18245082 PMC2431021

[ref39] Di Rita A. D'Acunzo P. Simula L. Campello S. Strappazzon F. Cecconi F. (2018). AMBRA1-mediated mitophagy counteracts oxidative stress and apoptosis induced by neurotoxicity in human neuroblastoma SH-SY5Y cells. Front. Cell. Neurosci. 12:92. doi: 10.3389/fncel.2018.00092, PMID: 29755319 PMC5932353

[ref40] Dilberger B. Baumanns S. Schmitt F. Schmiedl T. Hardt M. Wenzel U.. (2019). Mitochondrial oxidative stress impairs energy metabolism and reduces stress resistance and longevity of *C. elegans*. Oxidative Med. Cell. Longev. 2019, 1–14. doi: 10.1155/2019/6840540, PMID: 31827694 PMC6885289

[ref41] Doxaki C. Palikaras K. (2021). Neuronal mitophagy: friend or foe? Front. Cell Dev. Biol. 8:1864. doi: 10.3389/fcell.2020.611938, PMID: 33537304 PMC7848077

[ref42] Duffy J. B. (2002). GAL4 system in *Drosophila*: a fly geneticist's Swiss army knife. Genesis 34, 1–15. doi: 10.1002/gene.10150, PMID: 12324939

[ref43] Durcan T. M. Fon E. A. (2015). The three ‘P’s of mitophagy: PARKIN, PINK1, and post-translational modifications. Genes Dev. 29, 989–999. doi: 10.1101/gad.262758.115, PMID: 25995186 PMC4441056

[ref44] Eisenberg T. Abdellatif M. Schroeder S. Primessnig U. Stekovic S. Pendl T.. (2016). Cardioprotection and lifespan extension by the natural polyamine spermidine. Nat. Med. 22, 1428–1438. doi: 10.1038/nm.4222, PMID: 27841876 PMC5806691

[ref45] Eisenberg T. Knauer H. Schauer A. Büttner S. Ruckenstuhl C. Carmona-Gutierrez D.. (2009). Induction of autophagy by spermidine promotes longevity. Nat. Cell Biol. 11, 1305–1314. doi: 10.1038/ncb1975, PMID: 19801973

[ref46] Eiyama A. Okamoto K. (2015). PINK1/Parkin-mediated mitophagy in mammalian cells. Curr. Opin. Cell Biol. 33, 95–101. doi: 10.1016/j.ceb.2015.01.002 25697963

[ref47] Fan T. S. Liu S. C. Wu R. M. (2021). Alpha-synuclein and cognitive decline in Parkinson disease. Life 11:1239. doi: 10.3390/life11111239, PMID: 34833115 PMC8625417

[ref48] Feany M. B. Bender W. W. (2000). A *Drosophila* model of Parkinson's disease. Nature 404, 394–398. doi: 10.1038/35006074 10746727

[ref49] Federico A. Cardaioli E. da Pozzo P. Formichi P. Gallus G. N. Radi E. (2012). Mitochondria, oxidative stress and neurodegeneration. J. Neurol. Sci. 322, 254–262. doi: 10.1016/j.jns.2012.05.030 22669122

[ref50] Fernandez-Moreno M. A.. (2007). Drosophila melanogaster as a model system to study mitochondrial biology. Methods Mol. Biol. 372, 33–49. doi: 10.1007/978-1-59745-365-3_3, PMID: 18314716 PMC4876951

[ref51] Filosto M. Scarpelli M. Cotelli M. S. Vielmi V. Todeschini A. Gregorelli V.. (2011). The role of mitochondria in neurodegenerative diseases. J. Neurol. 258, 1763–1774. doi: 10.1007/s00415-011-6104-z 21604203

[ref52] Ganguly U. Chakrabarti S. S. Kaur U. Mukherjee A. Chakrabarti S. (2018). Alpha-synuclein, proteotoxicity and Parkinson's disease: search for neuroprotective therapy. Curr. Neuropharmacol. 16, 1086–1097. doi: 10.2174/1570159X15666171129100944, PMID: 29189163 PMC6120113

[ref53] Gan-Or Z. Dion P. A. Rouleau G. A. (2015). Genetic perspective on the role of the autophagy-lysosome pathway in Parkinson disease. Autophagy 11, 1443–1457. doi: 10.1080/15548627.2015.1067364, PMID: 26207393 PMC4590678

[ref54] Gan-Or Z. Liong C. Alcalay R. N. (2018). GBA-associated Parkinson’s disease and other synucleinopathies. Curr. Neurol. Neurosci. Rep. 62, 141–147. doi: 10.1007/s11910-018-0860-4 29884970

[ref55] Gatliff J. East D. Crosby J. Abeti R. Harvey R. Craigen W.. (2014). TSPO interacts with VDAC1 and triggers a ROS-mediated inhibition of mitochondrial quality control. Autophagy 10, 2279–2296. doi: 10.4161/15548627.2014.991665, PMID: 25470454 PMC4502750

[ref56] Geisler S. Holmström K. M. Skujat D. Fiesel F. C. Rothfuss O. C. Kahle P. J.. (2010). PINK1/Parkin-mediated mitophagy is dependent on VDAC1 and p62/SQSTM1. Nat. Cell Biol. 12, 119–131. doi: 10.1038/ncb2012, PMID: 20098416

[ref57] Georgakopoulos N. D. Wells G. Campanella M. (2017). The pharmacological regulation of cellular mitophagy. Nat. Chem. Biol. 13, 136–146. doi: 10.1038/nchembio.2287 28103219

[ref58] Ghodsi H. Rahimi H. R. Aghili S. M. Saberi A. Shoeibi A. (2022). Evaluation of curcumin as add-on therapy in patients with Parkinson's disease: a pilot randomized, triple-blind, placebo-controlled trial. Clin. Neurol. Neurosurg. 218:107300. doi: 10.1016/j.clineuro.2022.107300, PMID: 35636380

[ref59] Ghosh N. Das A. Biswas N. Gnyawali S. Singh K. Gorain M.. (2020). Urolithin a augments angiogenic pathways in skeletal muscle by bolstering NAD+ and SIRT1. Sci. Rep. 10, 1–13. doi: 10.1038/s41598-020-76564-7, PMID: 33214614 PMC7678835

[ref60] Ghosh I. Sankhe R. Mudgal J. Arora D. Nampoothiri M. (2020). Spermidine, an autophagy inducer, as a therapeutic strategy in neurological disorders. Neuropeptides 83:102083. doi: 10.1016/j.npep.2020.102083, PMID: 32873420

[ref61] Greene J. C. Whitworth A. J. Kuo I. Andrews L. A. Feany M. B. Pallanck L. J. (2003). Mitochondrial pathology and apoptotic muscle degeneration in *Drosophila* parkin mutants. Proc. Natl. Acad. Sci. 100, 4078–4083. doi: 10.1073/pnas.0737556100, PMID: 12642658 PMC153051

[ref62] Grünewald A. Kumar K. R. Sue C. M. (2019). New insights into the complex role of mitochondria in Parkinson’s disease. Prog. Neurobiol. 177, 73–93. doi: 10.1016/j.pneurobio.2018.09.003, PMID: 30219247

[ref63] Hanna R. A. Quinsay M. N. Orogo A. M. Giang K. Rikka S. Gustafsson Å. B. (2012). Microtubule-associated protein 1 light chain 3 (LC3) interacts with Bnip3 protein to selectively remove endoplasmic reticulum and mitochondria via autophagy. J. Biol. Chem. 287, 19094–19104. doi: 10.1074/jbc.M111.322933, PMID: 22505714 PMC3365942

[ref64] Harper J. W. Ordureau A. Heo J.-M. (2018). Building and decoding ubiquitin chains for mitophagy. Nat. Rev. Mol. Cell Biol. 19, 93–108. doi: 10.1038/nrm.2017.129, PMID: 29358684

[ref65] Hayashi T. Ishimori C. Takahashi-Niki K. Taira T. Kim Y. C. Maita H.. (2009). DJ-1 binds to mitochondrial complex I and maintains its activity. Biochem. Biophys. Res. Commun. 390, 667–672. doi: 10.1016/j.bbrc.2009.10.025, PMID: 19822128

[ref66] Heo J. Y. Park J. H. Kim S. J. Seo K. S. Han J. S. Lee S. H.. (2012). DJ-1 null dopaminergic neuronal cells exhibit defects in mitochondrial function and structure: involvement of mitochondrial complex I assembly. PLoS One 7:e32629. doi: 10.1371/journal.pone.0032629, PMID: 22403686 PMC3293835

[ref67] Hewitt V. L. Whitworth A. J. (2017). Mechanisms of Parkinson's disease: lessons from *Drosophila*. Curr. Top. Dev. Biol. 121, 173–200. doi: 10.1016/bs.ctdb.2016.07.005 28057299

[ref68] Hewlings S. J. Kalman D. S. (2017). Curcumin: a review of its effects on human health. Foods 6:92. doi: 10.3390/foods6100092, PMID: 29065496 PMC5664031

[ref69] Hisahara S. Shimohama S., Toxin-induced and genetic animal models of Parkinson's disease. Parkinson’s Dis., 2011. 2011, 2011, 1–14, doi: 10.4061/2011/951709, PMID: . 21234368 PMC3014721

[ref70] Hosseinpour-Moghaddam K. Caraglia M. Sahebkar A. (2018). Autophagy induction by trehalose: molecular mechanisms and therapeutic impacts. J. Cell. Physiol. 233, 6524–6543. doi: 10.1002/jcp.26583, PMID: 29663416 PMC13482534

[ref71] Hu D. Xie F. Xiao Y. Lu C. Zhong J. Huang D.. (2021). Metformin: a potential candidate for targeting aging mechanisms. Aging Dis. 12, 480–493. doi: 10.14336/AD.2020.0702, PMID: 33815878 PMC7990352

[ref72] Irrcher I. Aleyasin H. Seifert E. L. Hewitt S. J. Chhabra S. Phillips M.. (2010). Loss of the Parkinson's disease-linked gene DJ-1 perturbs mitochondrial dynamics. Hum. Mol. Genet. 19, 3734–3746. doi: 10.1093/hmg/ddq288, PMID: 20639397

[ref73] Jayatunga D. P. W. Hone E. Khaira H. Lunelli T. Singh H. Guillemin G. J.. (2021). Therapeutic potential of mitophagy-inducing microflora metabolite, Urolithin a for Alzheimer’s disease. Nutrients 13:3744. doi: 10.3390/nu13113744, PMID: 34836000 PMC8617978

[ref74] Jia S. Xu X. Zhou S. Chen Y. Ding G. Cao L. (2019). Fisetin induces autophagy in pancreatic cancer cells via endoplasmic reticulum stress-and mitochondrial stress-dependent pathways. Cell Death Dis. 10, 1–15. doi: 10.1038/s41419-019-1366-y PMC6374379 30760707

[ref75] Jin S. M. Youle R. J. (2012). PINK1-and Parkin-mediated mitophagy at a glance. J. Cell Sci. 125, 795–799. doi: 10.1242/jcs.093849, PMID: 22448035 PMC3656616

[ref76] Joselin A. P. Hewitt S. J. Callaghan S. M. Kim R. H. Chung Y. H. Mak T. W.. (2012). ROS-dependent regulation of Parkin and DJ-1 localization during oxidative stress in neurons. Hum. Mol. Genet. 21, 4888–4903. doi: 10.1093/hmg/dds325, PMID: 22872702

[ref77] Kampkötter A. Gombitang Nkwonkam C. Zurawski R. F. Timpel C. Chovolou Y. Wätjen W.. (2007). Effects of the flavonoids kaempferol and fisetin on thermotolerance, oxidative stress and FoxO transcription factor DAF-16 in the model organism Caenorhabditis elegans. Arch. Toxicol. 81, 849–858. doi: 10.1007/s00204-007-0215-4, PMID: 17551714

[ref78] Kampkötter A. Timpel C. Zurawski R. F. Ruhl S. Chovolou Y. Proksch P.. (2008). Increase of stress resistance and lifespan of Caenorhabditis elegans by quercetin. Comp. Biochem. Physiol. B: Biochem. Mol. Biol. 149, 314–323. doi: 10.1016/j.cbpb.2007.10.004, PMID: 18024103

[ref79] Kao C.-L. Chen L. K. Chang Y. L. Yung M. C. Hsu C. C. Chen Y. C.. (2010). Resveratrol protects human endothelium from H2O2-induced oxidative stress and senescence via SirT1 activation. J. Atheroscler. Thromb. 17, 970–979. doi: 10.5551/jat.4333, PMID: 20644332

[ref80] Karpinar D. P. Balija M. B. G. Kügler S. Opazo F. Rezaei-Ghaleh N. Wender N.. (2009). Pre-fibrillar α-synuclein variants with impaired β-structure increase neurotoxicity in Parkinson's disease models. EMBO J. 28, 3256–3268. doi: 10.1038/emboj.2009.257, PMID: 19745811 PMC2771093

[ref81] Kasture A. S. Hummel T. Sucic S. Freissmuth M. (2018). Big lessons from tiny flies: *Drosophila melanogaster* as a model to explore dysfunction of dopaminergic and serotonergic neurotransmitter systems. Int. J. Mol. Sci. 19:1788. doi: 10.3390/ijms19061788, PMID: 29914172 PMC6032372

[ref82] Kenwood B.M. Brookside NJ, Identification and design of a novel family mitochondrial protonophores for bioenergetics analysis and the treatment of ischemia-reperfusion injury. 2014.

[ref83] Khadrawy Y. A. Salem A. M. el-Shamy K. A. Ahmed E. K. Fadl N. N. Hosny E. N. (2017). Neuroprotective and therapeutic effect of caffeine on the rat model of Parkinson's disease induced by rotenone. J. Diet. Suppl. 14, 553–572. doi: 10.1080/19390211.2016.1275916, PMID: 28301304

[ref84] Khair S. B. A. Dhanushkodi N. R. Ardah M. T. Chen W. Yang Y. Haque M. E. (2018). Silencing of glucocerebrosidase gene in *Drosophila* enhances the aggregation of Parkinson's disease associated α-synuclein mutant A53T and affects locomotor activity. Front. Neurosci. 12:81. doi: 10.3389/fnins.2018.00081, PMID: 29503608 PMC5820349

[ref85] Khalifeh M. Barreto G. E. Sahebkar A. (2021). Therapeutic potential of trehalose in neurodegenerative diseases: the knowns and unknowns. Neural Regen. Res. 16, 2026–2027. doi: 10.4103/1673-5374.308085, PMID: 33642389 PMC8343311

[ref86] Kirienko N. V. Ausubel F. M. Ruvkun G. (2015). Mitophagy confers resistance to siderophore-mediated killing by Pseudomonas aeruginosa. Proc. Natl. Acad. Sci. 112, 1821–1826. doi: 10.1073/pnas.1424954112, PMID: 25624506 PMC4330731

[ref87] Kitada T. Asakawa S. Hattori N. Matsumine H. Yamamura Y. Minoshima S.. (1998). Mutations in the parkin gene cause autosomal recessive juvenile parkinsonism. Nature 392, 605–608. doi: 10.1038/33416, PMID: 9560156

[ref88] Kondapalli C. Kazlauskaite A. Zhang N. Woodroof H. I. Campbell D. G. Gourlay R.. (2012). PINK1 is activated by mitochondrial membrane potential depolarization and stimulates Parkin E3 ligase activity by phosphorylating serine 65. Open Biol. 2:120080. doi: 10.1098/rsob.120080, PMID: 22724072 PMC3376738

[ref89] Kropp P. A. Wu J. Reidy M. Shrestha S. Rhodehouse K. Rogers P.. (2021). Allele-specific mitochondrial stress induced by multiple mitochondrial dysfunctions syndrome 1 pathogenic mutations modeled in *Caenorhabditis elegans*. PLoS Genet. 17:e1009771. doi: 10.1371/journal.pgen.1009771, PMID: 34449775 PMC8428684

[ref90] Lagouge M. Argmann C. Gerhart-Hines Z. Meziane H. Lerin C. Daussin F.. (2006). Resveratrol improves mitochondrial function and protects against metabolic disease by activating SIRT1 and PGC-1α. Cells 127, 1109–1122. doi: 10.1016/j.cell.2006.11.013, PMID: 17112576

[ref91] Lazarou M. Sliter D. A. Kane L. A. Sarraf S. A. Wang C. Burman J. L.. (2015). The ubiquitin kinase PINK1 recruits autophagy receptors to induce mitophagy. Nature 524, 309–314. doi: 10.1038/nature14893, PMID: 26266977 PMC5018156

[ref92] Lee S. B. Kim W. Lee S. Chung J. (2007). Loss of LRRK2/PARK8 induces degeneration of dopaminergic neurons in *Drosophila*. Biochem. Biophys. Res. Commun. 358, 534–539. PMID: 17498648 10.1016/j.bbrc.2007.04.156

[ref93] Lee Y. Lee H. Y. Hanna R. A. Gustafsson Å. B. (2011). Mitochondrial autophagy by Bnip3 involves Drp1-mediated mitochondrial fission and recruitment of Parkin in cardiac myocytes. Am. J. Phys. Heart Circ. Phys. 301, H1924–H1931. doi: 10.1152/ajpheart.00368.2011, PMID: 21890690 PMC3213962

[ref94] Lee J.-E. Yoon S. S. Moon E.-Y. (2019). Curcumin-induced autophagy augments its antitumor effect against A172 human glioblastoma cells. Biomol. Ther. 27, 484–491. doi: 10.4062/biomolther.2019.107, PMID: 31405268 PMC6720530

[ref95] Li Q. Zhang T. Wang J. Zhang Z. Zhai Y. Yang G. Y.. (2014). Rapamycin attenuates mitochondrial dysfunction via activation of mitophagy in experimental ischemic stroke. Biochem. Biophys. Res. Commun. 444, 182–188. doi: 10.1016/j.bbrc.2014.01.032, PMID: 24440703

[ref96] Liao V. H.-C. Yu C. W. Chu Y. J. Li W. H. Hsieh Y. C. Wang T. T. (2011). Curcumin-mediated lifespan extension in *Caenorhabditis elegans*. Mech. Ageing Dev. 132, 480–487. doi: 10.1016/j.mad.2011.07.008, PMID: 21855561

[ref97] Lin T.-K. Chen S. D. Chuang Y. C. Lin H. Y. Huang C. R. Chuang J. H.. (2014). Resveratrol partially prevents rotenone-induced neurotoxicity in dopaminergic SH-SY5Y cells through induction of heme oxygenase-1 dependent autophagy. Int. J. Mol. Sci. 15, 1625–1646. doi: 10.3390/ijms15011625, PMID: 24451142 PMC3907890

[ref98] Lin X. Wen X. Wei Z. Guo K. Shi F. Huang T.. (2021). Vitamin K2 protects against Aβ42-induced neurotoxicity by activating autophagy and improving mitochondrial function in *Drosophila*. Neuroreport 32, 431–437. doi: 10.1097/WNR.0000000000001599, PMID: 33788812 PMC8016515

[ref99] Lindsley D. L. Zimm G. G. (2012). The genome of Drosophila melanogaster. San Diego, CA: Harcourt Brace Jovanovich. Academic press.

[ref100] Liu S. D’Amico D. Shankland E. Bhayana S. Garcia J. M. Aebischer P.. (2022). Effect of urolithin a supplementation on muscle endurance and mitochondrial health in older adults: a randomized clinical trial. JAMA Netw. Open 5, e2144279–e2144279. doi: 10.1001/jamanetworkopen.2021.44279, PMID: 35050355 PMC8777576

[ref101] Liu L. Feng D. Chen G. Chen M. Zheng Q. Song P.. (2012). Mitochondrial outer-membrane protein FUNDC1 mediates hypoxia-induced mitophagy in mammalian cells. Nat. Cell Biol. 14, 177–185. doi: 10.1038/ncb2422, PMID: 22267086

[ref102] Liu J. Liu W. Li R. Yang H. (2019). Mitophagy in Parkinson's disease: from pathogenesis to treatment. Cells 8:3055. doi: 10.3390/cells8070712, PMID: 31336937 PMC6678174

[ref103] Liu L. Sakakibara K. Chen Q. Okamoto K. (2014). Receptor-mediated mitophagy in yeast and mammalian systems. Cell Res. 24, 787–795. doi: 10.1038/cr.2014.75, PMID: 24903109 PMC4085769

[ref104] Liu Z. Wang X. Yu Y. Li X. Wang T. Jiang H.. (2008). A *Drosophila* model for LRRK2-linked parkinsonism. Proc. Natl. Acad. Sci. 105, 2693–2698. doi: 10.1073/pnas.0708452105, PMID: 18258746 PMC2268198

[ref105] Lou G. Palikaras K. Lautrup S. Scheibye-Knudsen M. Tavernarakis N. Fang E. F. (2020). Mitophagy and neuroprotection. Trends Mol. Med. 26, 8–20. doi: 10.1016/j.molmed.2019.07.002 31375365

[ref106] Luan P. D’Amico D. Andreux P. A. Laurila P. P. Wohlwend M. Li H.. (2021). Urolithin A improves muscle function by inducing mitophagy in muscular dystrophy. Sci. Transl. Med. 13:eabb0319. doi: 10.1126/scitranslmed.abb0319, PMID: 33827972

[ref107] Lucas J. I. Marín I. (2007). A new evolutionary paradigm for the Parkinson disease gene DJ-1. Mol. Biol. Evol. 24, 551–561. PMID: 17138626 10.1093/molbev/msl186

[ref108] Madeo F. Eisenberg T. Büttner S. Ruckenstuhl C. Kroemer G. (2010). Spermidine: a novel autophagy inducer and longevity elixir. Autophagy 6, 160–162. doi: 10.4161/auto.6.1.10600, PMID: 20110777

[ref109] Maor G. Cabasso O. Krivoruk O. Rodriguez J. Steller H. Segal D.. (2016). The contribution of mutant GBA to the development of Parkinson disease in *Drosophila*. Hum. Mol. Genet. 25, 2712–2727. PMID: 27162249 10.1093/hmg/ddw129 PMC6390410

[ref110] Markaki M. Palikaras K. Tavernarakis N. (2018). Novel insights into the anti-aging role of mitophagy. Int. Rev. Cell Mol. Biol. 340, 169–208. doi: 10.1016/bs.ircmb.2018.05.005, PMID: 30072091

[ref111] Maruzs T. Simon-Vecsei Z. Kiss V. Csizmadia T. Juhász G. (2019). On the fly: recent progress on autophagy and aging in *Drosophila*. Front. Cell Dev. Biol. 7:140. doi: 10.3389/fcell.2019.00140, PMID: 31396511 PMC6667644

[ref112] McWilliams T. G. Muqit M. M. (2017). PINK1 and Parkin: emerging themes in mitochondrial homeostasis. Curr. Opin. Cell Biol. 45, 83–91. doi: 10.1016/j.ceb.2017.03.013, PMID: 28437683

[ref113] McWilliams T. G. Prescott A. R. Montava-Garriga L. Ball G. Singh F. Barini E.. (2018). Basal mitophagy occurs independently of PINK1 in mouse tissues of high metabolic demand. Cell Metab. 27:e5, 439–449.e5. doi: 10.1016/j.cmet.2017.12.008, PMID: 29337137 PMC5807059

[ref114] Meissner C. Lorenz H. Hehn B. Lemberg M. K. (2015). Intramembrane protease PARL defines a negative regulator of PINK1-and PARK2/Parkin-dependent mitophagy. Autophagy 11, 1484–1498. doi: 10.1080/15548627.2015.1063763, PMID: 26101826 PMC4590680

[ref115] Menzies F. M. Yenisetti S. C. Min K.-T. (2005). Roles of *Drosophila* DJ-1 in survival of dopaminergic neurons and oxidative stress. Curr. Biol. 15, 1578–1582. doi: 10.1016/j.cub.2005.07.036, PMID: 16139214

[ref116] Meulener M. Whitworth A. J. Armstrong-Gold C. E. Rizzu P. Heutink P. Wes P. D.. (2005). Drosophila DJ-1 mutants are selectively sensitive to environmental toxins associated with Parkinson’s disease. Curr. Biol. 15, 1572–1577. doi: 10.1016/j.cub.2005.07.064, PMID: 16139213

[ref117] Meulener M. C. Xu K. Thomson L. Ischiropoulos H. Bonini N. M. (2006). Mutational analysis of DJ-1 in *Drosophila* implicates functional inactivation by oxidative damage and aging. Proc. Natl. Acad. Sci. 103, 12517–12522. doi: 10.1073/pnas.0601891103, PMID: 16894167 PMC1533799

[ref118] Miller S. Muqit M. M. K. (2019). Therapeutic approaches to enhance PINK1/Parkin mediated mitophagy for the treatment of Parkinson's disease. Neurosci. Lett. 705, 7–13. doi: 10.1016/j.neulet.2019.04.029, PMID: 30995519

[ref119] Miller-Fleming L. Olin-Sandoval V. Campbell K. Ralser M. (2015). Remaining mysteries of molecular biology: the role of polyamines in the cell. J. Mol. Biol. 427, 3389–3406. doi: 10.1016/j.jmb.2015.06.020, PMID: 26156863

[ref120] Misgeld T. Schwarz T. L. (2017). Mitostasis in neurons: maintaining mitochondria in an extended cellular architecture. Neuron 96, 651–666. doi: 10.1016/j.neuron.2017.09.055, PMID: 29096078 PMC5687842

[ref121] Moisoi N. Fedele V. Edwards J. Martins L. M. (2014). Loss of PINK1 enhances neurodegeneration in a mouse model of Parkinson's disease triggered by mitochondrial stress. Neuropharmacology 77, 350–357. doi: 10.1016/j.neuropharm.2013.10.009, PMID: 24161480 PMC3878764

[ref122] Molina-Mateo D. Fuenzalida-Uribe N. Hidalgo S. Molina-Fernández C. Abarca J. Zárate R. V.. (2017). Characterization of a presymptomatic stage in a *Drosophila* Parkinson's disease model: unveiling dopaminergic compensatory mechanisms. Biochim. Biophys. Acta - Mol. Basis Dis. 1863, 2882–2890. doi: 10.1016/j.bbadis.2017.07.013, PMID: 28716706

[ref123] Momtazi-Borojeni A. A. Ghasemi F. Hesari A. Majeed M. Caraglia M. Sahebkar A. (2018). Anti-cancer and radio-sensitizing effects of curcumin in nasopharyngeal carcinoma. Curr. Pharm. Des. 24, 2121–2128. doi: 10.2174/1381612824666180522105202, PMID: 29788875

[ref124] Morais V. A. Haddad D. Craessaerts K. de Bock P. J. Swerts J. Vilain S.. (2014). PINK1 loss-of-function mutations affect mitochondrial complex I activity via NdufA10 ubiquinone uncoupling. Science 344, 203–207. doi: 10.1126/science.1249161, PMID: 24652937

[ref125] Morales P. E. Arias-Durán C. Ávalos-Guajardo Y. Aedo G. Verdejo H. E. Parra V.. (2020). Emerging role of mitophagy in cardiovascular physiology and pathology. Mol. Asp. Med. 71:100822. doi: 10.1016/j.mam.2019.09.006, PMID: 31587811

[ref126] Moullan N. Mouchiroud L. Wang X. Ryu D. Williams E. G. Mottis A.. (2015). Tetracyclines disturb mitochondrial function across eukaryotic models: a call for caution in biomedical research. Cell Rep. 10, 1681–1691. doi: 10.1016/j.celrep.2015.02.034, PMID: 25772356 PMC4565776

[ref127] Muqit M. M. Feany M. B. (2002). Modelling neurodegenerative diseases in *Drosophila*: a fruitful approach? Nat. Rev. Neurosci. 3, 237–243. doi: 10.1038/nrn751, PMID: 11994755

[ref128] Murakawa T. Yamaguchi O. Hashimoto A. Hikoso S. Takeda T. Oka T.. (2015). Bcl-2-like protein 13 is a mammalian Atg32 homologue that mediates mitophagy and mitochondrial fragmentation. Nat. Commun. 6, 1–14. doi: 10.1038/ncomms8527, PMID: 26146385 PMC4501433

[ref129] Nagi M. Tanabe K. Nakayama H. Ueno K. Yamagoe S. Umeyama T.. (2016). Iron-depletion promotes mitophagy to maintain mitochondrial integrity in pathogenic yeast *Candida glabrata*. Autophagy 12, 1259–1271. doi: 10.1080/15548627.2016.1183080, PMID: 27347716 PMC4968229

[ref130] Naoi M. Wu Y. Shamoto-Nagai M. Maruyama W. (2019). Mitochondria in neuroprotection by phytochemicals: bioactive polyphenols modulate mitochondrial apoptosis system, function and structure. Int. J. Mol. Sci. 20:2451. doi: 10.3390/ijms20102451, PMID: 31108962 PMC6566187

[ref131] Narendra D. P. Jin S. M. Tanaka A. Suen D. F. Gautier C. A. Shen J.. (2010). PINK1 is selectively stabilized on impaired mitochondria to activate Parkin. PLoS Biol. 8:e1000298. doi: 10.1371/journal.pbio.1000298, PMID: 20126261 PMC2811155

[ref132] Narendra D. Kane L. A. Hauser D. N. Fearnley I. M. Youle R. J. (2010). p62/SQSTM1 is required for Parkin-induced mitochondrial clustering but not mitophagy; VDAC1 is dispensable for both. Autophagy 6, 1090–1106. doi: 10.4161/auto.6.8.13426, PMID: 20890124 PMC3359490

[ref133] Narendra D. Tanaka A. Suen D. F. Youle R. J. (2008). Parkin is recruited selectively to impaired mitochondria and promotes their autophagy. J. Cell Biol. 183, 795–803. doi: 10.1083/jcb.200809125, PMID: 19029340 PMC2592826

[ref134] Navarro J. A. Heßner S. Yenisetti S. C. Bayersdorfer F. Zhang L. Voigt A.. (2014). Analysis of dopaminergic neuronal dysfunction in genetic and toxin-induced models of Parkinson's disease in *Drosophila*. J. Neurochem. 131, 369–382. doi: 10.1111/jnc.12818, PMID: 25040725

[ref135] Naz F. Siddique Y. H. (2021). Drosophila melanogaster a versatile model of Parkinson’s disease. CNS & Neurological Disorders-Drug Targets (formerly Current Drug Targets-CNS & Neurological Disorders) 20, 487–530. doi: 10.2174/1871527320666210208125912, 33557742

[ref136] Ni Y.-Q. Liu Y.-S. (2021). New insights into the roles and mechanisms of spermidine in aging and age-related diseases. Aging Dis. 12, 1948–1963. doi: 10.14336/AD.2021.0603, PMID: 34881079 PMC8612618

[ref137] Palikaras K. Daskalaki I. Markaki M. Tavernarakis N. (2017). Mitophagy and age-related pathologies: development of new therapeutics by targeting mitochondrial turnover. Pharmacol. Ther. 178, 157–174. doi: 10.1016/j.pharmthera.2017.04.005, PMID: 28461251

[ref138] Palikaras K. Lionaki E. Tavernarakis N. (2015). Coordination of mitophagy and mitochondrial biogenesis during ageing in *C. elegans*. Nature 521, 525–528. doi: 10.1038/nature14300, PMID: 25896323

[ref139] Palikaras K. Lionaki E. Tavernarakis N. (2018a). Mechanisms of mitophagy in cellular homeostasis, physiology and pathology. Nat. Cell Biol. 20, 1013–1022. doi: 10.1038/s41556-018-0176-2 30154567

[ref140] Palikaras K. Princz A. Tavernarakis N. (2018b). Mitophagy modulators, Encyclopedia of Biomedical Gerontology, ed. Rattan, S. I.S.Elsevier: Academic Press, 2, 433–446.

[ref141] Palikaras K. Tavernarakis N. (2012). Mitophagy in neurodegeneration and aging. Front. Genet. 3:297. doi: 10.3389/fgene.2012.00297, PMID: 23267366 PMC3525948

[ref142] Palikaras K. Tavernarakis N. (2020). Regulation and roles of mitophagy at synapses. Mech. Ageing Dev. 187:111216. doi: 10.1016/j.mad.2020.111216, PMID: 32084458

[ref143] Pallauf K. Rimbach G. (2013). Autophagy, polyphenols and healthy ageing. Ageing Res. Rev. 12, 237–252. doi: 10.1016/j.arr.2012.03.008, PMID: 22504405

[ref144] Park Y. S. Choi S. E. Koh H. C. (2018). PGAM5 regulates PINK1/Parkin-mediated mitophagy via DRP1 in CCCP-induced mitochondrial dysfunction. Toxicol. Lett. 284, 120–128. doi: 10.1016/j.toxlet.2017.12.004, PMID: 29241732

[ref145] Park J. Kim S. Y. Cha G. H. Lee S. B. Kim S. Chung J. (2005). Drosophila DJ-1 mutants show oxidative stress-sensitive locomotive dysfunction. Gene 361, 133–139. doi: 10.1016/j.gene.2005.06.040, PMID: 16203113

[ref146] Park J. Lee S. B. Lee S. Kim Y. Song S. Kim S.. (2006). Mitochondrial dysfunction in *Drosophila* PINK1 mutants is complemented by parkin. Nature 441, 1157–1161. doi: 10.1038/nature04788, PMID: 16672980

[ref147] Patel C. Dadhaniya P. Hingorani L. Soni M. G. (2008). Safety assessment of pomegranate fruit extract: acute and subchronic toxicity studies. Food Chem. Toxicol. 46, 2728–2735. doi: 10.1016/j.fct.2008.04.035, PMID: 18571823

[ref148] Pickrell A. M. Youle R. J. (2015). The roles of PINK1, parkin, and mitochondrial fidelity in Parkinson’s disease. Neuron 85, 257–273. doi: 10.1016/j.neuron.2014.12.007, PMID: 25611507 PMC4764997

[ref149] Pillai V. B. Sundaresan N. R. Kim G. Gupta M. Rajamohan S. B. Pillai J. B.. (2010). Exogenous NAD blocks cardiac hypertrophic response via activation of the SIRT3-LKB1-AMP-activated kinase pathway. J. Biol. Chem. 285, 3133–3144. doi: 10.1074/jbc.M109.077271, PMID: 19940131 PMC2823454

[ref150] Polymeropoulos M. H. Lavedan C. Leroy E. Ide S. E. Dehejia A. Dutra A.. (1997). Mutation in the α-synuclein gene identified in families with Parkinson's disease. Science 276, 2045–2047. doi: 10.1126/science.276.5321.2045 9197268

[ref151] Qi Y. Qiu Q. Gu X. Tian Y. Zhang Y. (2016). ATM mediates spermidine-induced mitophagy via PINK1 and Parkin regulation in human fibroblasts. Sci. Rep. 6, 1–11. doi: 10.1038/srep24700, PMID: 27089984 PMC4835770

[ref152] Ramirez A. Heimbach A. Gründemann J. Stiller B. Hampshire D. Cid L. P.. (2006). Hereditary parkinsonism with dementia is caused by mutations in ATP13A2, encoding a lysosomal type 5 P-type ATPase. Nat. Genet. 38, 1184–1191. doi: 10.1038/ng1884, PMID: 16964263

[ref153] Redmann M. Benavides G. A. Berryhill T. F. Wani W. Y. Ouyang X. Johnson M. S.. (2017). Inhibition of autophagy with bafilomycin and chloroquine decreases mitochondrial quality and bioenergetic function in primary neurons. Redox Biol. 11, 73–81. doi: 10.1016/j.redox.2016.11.004, PMID: 27889640 PMC5124357

[ref154] Redmann M. Dodson M. Boyer-Guittaut M. Darley-Usmar V. Zhang J. (2014). Mitophagy mechanisms and role in human diseases. Int. J. Biochem. Cell Biol. 53, 127–133. doi: 10.1016/j.biocel.2014.05.010, PMID: 24842106 PMC4111979

[ref155] Richter U. Lahtinen T. Marttinen P. Myöhänen M. Greco D. Cannino G.. (2013). A mitochondrial ribosomal and RNA decay pathway blocks cell proliferation. Curr. Biol. 23, 535–541. doi: 10.1016/j.cub.2013.02.019, PMID: 23453957

[ref156] Rogov V. V. Suzuki H. Marinković M. Lang V. Kato R. Kawasaki M.. (2017). Phosphorylation of the mitochondrial autophagy receptor nix enhances its interaction with LC3 proteins. Sci. Rep. 7, 1–12. doi: 10.1038/s41598-017-01258-6, PMID: 28442745 PMC5430633

[ref157] Romero-Garcia S. Prado-Garcia H. (2019). Mitochondrial calcium: transport and modulation of cellular processes in homeostasis and cancer. Int. J. Oncol. 54, 1155–1167. doi: 10.3892/ijo.2019.4696, PMID: 30720054

[ref158] Rosenbusch K. E. Kortholt A. (2016). Activation mechanism of LRRK2 and its cellular functions in Parkinson's disease. Parkinson's Dis. 2016, 1–8. doi: 10.1155/2016/7351985, PMID: 27293958 PMC4880697

[ref159] Roy B. Jackson G. R. (2014). Interactions between tau and α-synuclein augment neurotoxicity in a *Drosophila* model of Parkinson's disease. Hum. Mol. Genet. 23, 3008–3023. doi: 10.1093/hmg/ddu011, PMID: 24430504 PMC4014195

[ref160] Ryu D. Mouchiroud L. Andreux P. A. Katsyuba E. Moullan N. Nicolet-dit-Félix A. A.. (2016). Urolithin A induces mitophagy and prolongs lifespan in *C. elegans* and increases muscle function in rodents. Nat. Med. 22, 879–888. doi: 10.1038/nm.4132, PMID: 27400265

[ref161] Sanchez-Martinez A. Beavan M. Gegg M. E. Chau K.-Y. Whitworth A. J. Schapira A. H. V. (2016). Parkinson disease-linked GBA mutation effects reversed by molecular chaperones in human cell and fly models. Sci. Rep. 6, 1–12. doi: 10.1038/srep31380, PMID: 27539639 PMC4990939

[ref162] Schiavi A. Maglioni S. Palikaras K. Shaik A. Strappazzon F. Brinkmann V.. (2015). Iron-starvation-induced mitophagy mediates lifespan extension upon mitochondrial stress in *C. elegans*. Curr. Biol. 25, 1810–1822. doi: 10.1016/j.cub.2015.05.059, PMID: 26144971

[ref163] Schober A. (2004). Classic toxin-induced animal models of Parkinson’s disease: 6-OHDA and MPTP. Cell Tissue Res. 318, 215–224. doi: 10.1007/s00441-004-0938-y, PMID: 15503155

[ref164] Schwarz C. Stekovic S. Wirth M. Benson G. Royer P. Sigrist S. J.. (2018). Safety and tolerability of spermidine supplementation in mice and older adults with subjective cognitive decline. Aging (Albany NY) 10, 19–33. doi: 10.18632/aging.101354, PMID: 29315079 PMC5807086

[ref165] Schweers R. L. Zhang J. Randall M. S. Loyd M. R. Li W. Dorsey F. C.. (2007). NIX is required for programmed mitochondrial clearance during reticulocyte maturation. Proc. Natl. Acad. Sci. 104, 19500–19505. doi: 10.1073/pnas.0708818104, PMID: 18048346 PMC2148318

[ref166] Sebastián D. Sorianello E. Segalés J. Irazoki A. Ruiz-Bonilla V. Sala D.. (2016). Mfn2 deficiency links age-related sarcopenia and impaired autophagy to activation of an adaptive mitophagy pathway. EMBO J. 35, 1677–1693. doi: 10.15252/embj.201593084, PMID: 27334614 PMC4969577

[ref167] Sekine S. Youle R. J. (2018). PINK1 import regulation; a fine system to convey mitochondrial stress to the cytosol. BMC Biol. 16, 1–12. doi: 10.1186/s12915-017-0470-7 29325568 PMC5795276

[ref168] Shakeri A. Cicero A. F. G. Panahi Y. Mohajeri M. Sahebkar A. (2019). Curcumin: a naturally occurring autophagy modulator. J. Cell. Physiol. 234, 5643–5654. doi: 10.1002/jcp.27404, PMID: 30239005

[ref169] Shanmugam M. K. Rane G. Kanchi M. M. Arfuso F. Chinnathambi A. Zayed M. E.. (2015). The multifaceted role of curcumin in cancer prevention and treatment. Molecules 20, 2728–2769. doi: 10.3390/molecules20022728, PMID: 25665066 PMC6272781

[ref170] Sherer T. B. Betarbet R. Testa C. M. Seo B. B. Richardson J. R. Kim J. H.. (2003). Mechanism of toxicity in rotenone models of Parkinson's disease. J. Neurosci. 23, 10756–10764. doi: 10.1523/JNEUROSCI.23-34-10756.2003, PMID: 14645467 PMC6740985

[ref171] Shi M.-M. Shi C.-H. Xu Y.-M. (2017). Rab GTPases: the key players in the molecular pathway of Parkinson’s disease. Front. Cell. Neurosci. 11:81. doi: 10.3389/fncel.2017.00081, PMID: 28400718 PMC5369176

[ref172] Shi W. Xiao D. Wang L. Dong L.-H. Yan Z.-X. Shen Z.-X.. (2012). Therapeutic metformin/AMPK activation blocked lymphoma cell growth via inhibition of mTOR pathway and induction of autophagy. Cell Death Dis. 3, e275–e275. doi: 10.1038/cddis.2012.13 22378068 PMC3317343

[ref173] Singh F. Ganley I. G. (2021). Parkinson's disease and mitophagy: an emerging role for LRRK2. Biochem. Soc. Trans. 49, 551–562. doi: 10.1042/BST20190236, PMID: 33769432 PMC8106497

[ref174] Singh A. P. Singh R. Verma S. S. Rai V. Kaschula C. H. Maiti P.. (2019). Health benefits of resveratrol: evidence from clinical studies. Med. Res. Rev. 39, 1851–1891. doi: 10.1002/med.21565, PMID: 30741437

[ref175] Southall T. D. Elliott D. A. Brand A. H. (2008). The GAL4 system: a versatile toolkit for gene expression in drosophila. Cold Spring Harb Protoc 2008:pdb.top49. doi: 10.1101/pdb.top49 21356876

[ref176] Srivastava P. Panda D. (2007). Rotenone inhibits mammalian cell proliferation by inhibiting microtubule assembly through tubulin binding. FEBS J. 274, 4788–4801. doi: 10.1111/j.1742-4658.2007.06004.x, PMID: 17697112

[ref177] Sun A. Y. Wang Q. Simonyi A. Sun G. Y. (2010). Resveratrol as a therapeutic agent for neurodegenerative diseases. Mol. Neurobiol. 41, 375–383. doi: 10.1007/s12035-010-8111-y, PMID: 20306310 PMC3076208

[ref178] Suzuki M. Fujikake N. Takeuchi T. Kohyama-Koganeya A. Nakajima K. Hirabayashi Y.. (2015). Glucocerebrosidase deficiency accelerates the accumulation of proteinase K-resistant α-synuclein and aggravates neurodegeneration in a drosophila model of Parkinson's disease. Hum. Mol. Genet. 24, 6675–6686. doi: 10.1093/hmg/ddv372, PMID: 26362253

[ref179] Tieu K. (2011). A guide to neurotoxic animal models of Parkinson’s disease. Cold Spring Harb. Perspect. Med. 1:a009316. doi: 10.1101/cshperspect.a009316, PMID: 22229125 PMC3234449

[ref180] Tong D. Hill J. A. (2017). Spermidine promotes cardioprotective autophagy. Circ. Res. 120, 1229–1231. doi: 10.1161/CIRCRESAHA.117.310603, PMID: 28408448 PMC5411858

[ref181] Trempe J.-F. Sauvé V. Grenier K. Seirafi M. Tang M. Y. Ménade M.. (2013). Structure of parkin reveals mechanisms for ubiquitin ligase activation. Science 340, 1451–1455. doi: 10.1126/science.1237908, PMID: 23661642

[ref182] Trinh K. Andrews L. Krause J. Hanak T. Lee D. Gelb M.. (2010). Decaffeinated coffee and nicotine-free tobacco provide neuroprotection in *Drosophila* models of Parkinson's disease through an NRF2-dependent mechanism. J. Neurosci. 30, 5525–5532. doi: 10.1523/JNEUROSCI.4777-09.2010, PMID: 20410106 PMC3842467

[ref183] Uversky V. N. (2004). Neurotoxicant-induced animal models of Parkinson’s disease: understanding the role of rotenone, maneb and paraquat in neurodegeneration. Cell Tissue Res. 318, 225–241. doi: 10.1007/s00441-004-0937-z, PMID: 15258850

[ref184] Valente E. M. Abou-Sleiman P. M. Caputo V. Muqit M. M. K. Harvey K. Gispert S.. (2004). Hereditary early-onset Parkinson's disease caused by mutations in PINK1. Science 304, 1158–1160. doi: 10.1126/science.1096284, PMID: 15087508

[ref185] Van der Merwe C. van Dyk H. C. Engelbrecht L. van der Westhuizen F. H. Kinnear C. Loos B.. (2017). Curcumin rescues a PINK1 knock down SH-SY5Y cellular model of Parkinson's disease from mitochondrial dysfunction and cell death. Mol. Neurobiol. 54, 2752–2762. doi: 10.1007/s12035-016-9843-0, PMID: 27003823

[ref186] Vara-Perez M. Felipe-Abrio B. Agostinis P. (2019). Mitophagy in cancer: a tale of adaptation. Cells 8:493. doi: 10.3390/cells8050493, PMID: 31121959 PMC6562743

[ref187] Velayati A. Yu W. H. Sidransky E. (2010). The role of glucocerebrosidase mutations in Parkinson disease and Lewy body disorders. Curr. Neurol. Neurosci. Rep. 10, 190–198. doi: 10.1007/s11910-010-0102-x, PMID: 20425034 PMC3529411

[ref188] Villa E. Proïcs E. Rubio-Patiño C. Obba S. Zunino B. Bossowski J. P.. (2017). Parkin-independent mitophagy controls chemotherapeutic response in cancer cells. Cell Rep. 20, 2846–2859. doi: 10.1016/j.celrep.2017.08.087, PMID: 28930681

[ref189] Villanueva Paz M. Cotán D. Garrido-Maraver J. Cordero M. D. Oropesa-Ávila M. de la Mata M.. (2016). Targeting autophagy and mitophagy for mitochondrial diseases treatment. Expert Opin. Ther. Targets 20, 487–500. doi: 10.1517/14728222.2016.1101068 26523761

[ref190] Vos M. Esposito G. Edirisinghe J. N. Vilain S. Haddad D. M. Slabbaert J. R.. (2012). Vitamin K2 is a mitochondrial electron carrier that rescues pink1 deficiency. Science 336, 1306–1310. doi: 10.1126/science.1218632, PMID: 22582012

[ref191] Wakabayashi K. Takahashi H. (2007). Pathology of familial Parkinson's disease. Brain Nerve 59, 851–864. PMID: 17713121

[ref192] Wallings R. Manzoni C. Bandopadhyay R. (2015). Cellular processes associated with LRRK 2 function and dysfunction. FEBS J. 282, 2806–2826. doi: 10.1111/febs.13305, PMID: 25899482 PMC4522467

[ref193] Wang W.-W. Han R. He H. J. Li J. Chen S. Y. Gu Y.. (2021). Administration of quercetin improves mitochondria quality control and protects the neurons in 6-OHDA-lesioned Parkinson's disease models. Aging (Albany NY) 13, 11738–11751. doi: 10.18632/aging.202868, PMID: 33878030 PMC8109056

[ref194] Wang T. Hay J. C. (2015). Alpha-synuclein toxicity in the early secretory pathway: how it drives neurodegeneration in Parkinsons disease. Front. Neurosci. 9:433. doi: 10.3389/fnins.2015.00433, PMID: 26617485 PMC4641903

[ref195] Wang H.-S. Toh J. Ho P. Tio M. Zhao Y. Tan E. K. (2014). In vivo evidence of pathogenicity of VPS35 mutations in the *Drosophila*. Mol. Brain 7, 1–6. doi: 10.1186/s13041-014-0073-y, PMID: 25288323 PMC4193144

[ref196] Wang W. Xu J. (2020). Curcumin attenuates cerebral ischemia-reperfusion injury through regulating mitophagy and preserving mitochondrial function. Curr. Neurovasc. Res. 17, 113–122. doi: 10.2174/1567202617666200225122620, PMID: 32096742

[ref197] Wang X. Zhang X. Huang Z. Wu D. Liu B. Zhang R.. (2016). Protons trigger mitochondrial flashes. Biophys. J. 111, 386–394. doi: 10.1016/j.bpj.2016.05.052, PMID: 27463140 PMC4968422

[ref198] Wauters F. Cornelissen T. Imberechts D. Martin S. Koentjoro B. Sue C.. (2020). LRRK2 mutations impair depolarization-induced mitophagy through inhibition of mitochondrial accumulation of RAB10. Autophagy 16, 203–222. doi: 10.1080/15548627.2019.1603548, PMID: 30945962 PMC6984591

[ref199] Whitworth A. J. Theodore D. A. Greene J. C. Beneš H. Wes P. D. Pallanck L. J. (2005). Increased glutathione S-transferase activity rescues dopaminergic neuron loss in a drosophila model of Parkinson's disease. Proc. Natl. Acad. Sci. 102, 8024–8029. doi: 10.1073/pnas.0501078102, PMID: 15911761 PMC1142368

[ref200] Wirth M. Benson G. Schwarz C. Köbe T. Grittner U. Schmitz D.. (2018). The effect of spermidine on memory performance in older adults at risk for dementia: a randomized controlled trial. Cortex 109, 181–188. doi: 10.1016/j.cortex.2018.09.014, PMID: 30388439

[ref201] Witt S. N. (2013). Molecular chaperones, alpha-synuclein, and neurodegeneration. Mol. Neurobiol. 47, 552–560. doi: 10.1007/s12035-012-8325-2, PMID: 22923346 PMC3537861

[ref202] Wong Y. C. Holzbaur E. L. (2015). Temporal dynamics of PARK2/parkin and OPTN/optineurin recruitment during the mitophagy of damaged mitochondria. Autophagy 11, 422–424. doi: 10.1080/15548627.2015.1009792, PMID: 25801386 PMC4502688

[ref203] Wu X. al-Amin M. Zhao C. An F. Wang Y. Huang Q.. (2020). Catechinic acid, a natural polyphenol compound, extends the lifespan of Caenorhabditis elegans via mitophagy pathways. Food Funct. 11, 5621–5634. doi: 10.1039/D0FO00694G, PMID: 32530444

[ref204] Wu M.-L. Li H. Yu L. J. Chen X. Y. Kong Q. Y. Song X.. (2014). Short-term resveratrol exposure causes in vitro and in vivo growth inhibition and apoptosis of bladder cancer cells. PLoS One 9:e89806. doi: 10.1371/journal.pone.0089806, PMID: 24587049 PMC3934942

[ref205] Wu Y. Li X. Zhu J. X. Xie W. le W. Fan Z.. (2011). Resveratrol-activated AMPK/SIRT1/autophagy in cellular models of Parkinson’s disease. Neurosignals 19, 163–174. doi: 10.1159/000328516, PMID: 21778691 PMC3699815

[ref206] Wu Y.-T. Tan H. L. Shui G. Bauvy C. Huang Q. Wenk M. R.. (2010). Dual role of 3-methyladenine in modulation of autophagy via different temporal patterns of inhibition on class I and III phosphoinositide 3-kinase. J. Biol. Chem. 285, 10850–10861. doi: 10.1074/jbc.M109.080796, PMID: 20123989 PMC2856291

[ref207] Wu Z. Wu A. Dong J. Sigears A. Lu B. (2018). Grape skin extract improves muscle function and extends lifespan of a *Drosophila* model of Parkinson's disease through activation of mitophagy. Exp. Gerontol. 113, 10–17. doi: 10.1016/j.exger.2018.09.014, PMID: 30248358 PMC12950264

[ref208] Yamano K. Youle R. J. (2013). PINK1 is degraded through the N-end rule pathway. Autophagy 9, 1758–1769. doi: 10.4161/auto.24633, PMID: 24121706 PMC4028335

[ref209] Yang Y. Gehrke S. Imai Y. Huang Z. Ouyang Y. Wang J. W.. (2006). Mitochondrial pathology and muscle and dopaminergic neuron degeneration caused by inactivation of *Drosophila* Pink1 is rescued by Parkin. Proc. Natl. Acad. Sci. 103, 10793–10798. doi: 10.1073/pnas.0602493103, PMID: 16818890 PMC1502310

[ref210] Yang D. Thomas J. M. Li T. Lee Y. Liu Z. Smith W. W. (2018). The drosophila hep pathway mediates Lrrk2-induced neurodegeneration. Biochem. Cell Biol. 96, 441–449. doi: 10.1139/bcb-2017-0262, PMID: 29268033 PMC6441734

[ref211] Yang X. Zhang M. Dai Y. Sun Y. Aman Y. Xu Y.. (2020). Spermidine inhibits neurodegeneration and delays aging via the PINK1-PDR1-dependent mitophagy pathway in *C. elegans*. Aging (Albany NY) 12, 16852–16866. doi: 10.18632/aging.103578, PMID: 32902411 PMC7521492

[ref212] Yin G. Lopes da Fonseca T. Eisbach S. E. Anduaga A. M. Breda C. Orcellet M. L.. (2014). α-Synuclein interacts with the switch region of Rab8a in a Ser129 phosphorylation-dependent manner. Neurobiol. Dis. 70, 149–161. doi: 10.1016/j.nbd.2014.06.018, PMID: 24983211

[ref213] Youle R. J. Narendra D. P. (2011). Mechanisms of mitophagy. Nat. Rev. Mol. Cell Biol. 12, 9–14. doi: 10.1038/nrm3028, PMID: 21179058 PMC4780047

[ref214] Yun J. Puri R. Yang H. Lizzio M. A. Wu C. Sheng Z. H.. (2014). MUL1 acts in parallel to the PINK1/parkin pathway in regulating mitofusin and compensates for loss of PINK1/parkin. elife 3:e01958. doi: 10.7554/eLife.01958, PMID: 24898855 PMC4044952

[ref215] Zhang Y. Aisker G. Dong H. Halemahebai G. Zhang Y. Tian L. (2021). Urolithin A suppresses glucolipotoxicity-induced ER stress and TXNIP/NLRP3/IL-1β inflammation signal in pancreatic β cells by regulating AMPK and autophagy. Phytomedicine 93:153741. doi: 10.1016/j.phymed.2021.153741, PMID: 34656886

[ref216] Zhang L. Shimoji M. Thomas B. Moore D. J. Yu S. W. Marupudi N. I.. (2005). Mitochondrial localization of the Parkinson's disease related protein DJ-1: implications for pathogenesis. Hum. Mol. Genet. 14, 2063–2073. doi: 10.1093/hmg/ddi211, PMID: 15944198

[ref217] Zimmermann M. Reichert A. S. (2018). How to get rid of mitochondria: crosstalk and regulation of multiple mitophagy pathways. Biol. Chem. 399, 29–45. doi: 10.1515/hsz-2017-0206, PMID: 28976890

[ref218] Zimprich A. Biskup S. Leitner P. Lichtner P. Farrer M. Lincoln S.. (2004). Mutations in LRRK2 cause autosomal-dominant parkinsonism with pleomorphic pathology. Neuron 44, 601–607. doi: 10.1016/j.neuron.2004.11.005, PMID: 15541309

